# A general approach for detecting expressed mutations in AML cells using single cell RNA-sequencing

**DOI:** 10.1038/s41467-019-11591-1

**Published:** 2019-08-14

**Authors:** Allegra A. Petti, Stephen R. Williams, Christopher A. Miller, Ian T. Fiddes, Sridhar N. Srivatsan, David Y. Chen, Catrina C. Fronick, Robert S. Fulton, Deanna M. Church, Timothy J. Ley

**Affiliations:** 1 Division of Oncology, Washington University School of Medicine, St. Louis, MO USA; 2 McDonnell Genome Institute, Washington University School of Medicine, St. Louis, MO USA; 3 10x Genomics, Inc., Pleasanton, CA USA; 4 Division of Dermatology, Washington University School of Medicine, St. Louis, MO USA; 5 Inscripta, Inc., Boulder, CO USA; 6 Department of Genetics, Washington University School of Medicine, St. Louis, MO USA

**Keywords:** Cancer genomics, Acute myeloid leukaemia, Tumour heterogeneity, Data integration

## Abstract

Virtually all tumors are genetically heterogeneous, containing mutationally-defined subclonal cell populations that often have distinct phenotypes. Single-cell RNA-sequencing has revealed that a variety of tumors are also transcriptionally heterogeneous, but the relationship between expression heterogeneity and subclonal architecture is unclear. Here, we address this question in the context of Acute Myeloid Leukemia (AML) by integrating whole genome sequencing with single-cell RNA-sequencing (using the 10x Genomics Chromium Single Cell 5’ Gene Expression workflow). Applying this approach to five cryopreserved AML samples, we identify hundreds to thousands of cells containing tumor-specific mutations in each case, and use the results to distinguish AML cells (including normal-karyotype AML cells) from normal cells, identify expression signatures associated with subclonal mutations, and find cell surface markers that could be used to purify subclones for further study. This integrative approach for connecting genotype to phenotype is broadly applicable to any sample that is phenotypically and genetically heterogeneous.

## Abstract (web summary)

The advent of single-cell RNA sequencing has revealed significant transcriptional heterogeneity in cancer, but its relationship to genomic heterogeneity remains unclear. Focusing on acute myeloid leukemia samples, the authors describe a general approach for linking mutation-containing cells to their transcriptional phenotypes using single-cell RNA sequencing data.

## Introduction

Connecting genotype to phenotype at the single-cell level is widely appreciated as a central challenge in the analysis and interpretation of scRNA-seq data, with applications ranging from cell lineage tracing^1^ and eQTL discovery^2^, to the analysis of subclonal architecture in tumors^3–14^. In cancer, mutationally distinct subclones can differ with respect to key clinical properties such as drug sensitivity and growth rate, and this phenotypic diversity may contribute to drug resistance and tumor evolution^15^. However, it is currently difficult to purify individual subclones for use in downstream studies that address the biological basis of these differences. Meanwhile, a growing body of work has demonstrated that tumors are also transcriptionally heterogeneous, but it has been challenging to relate this epigenetic heterogeneity to genetic heterogeneity in individual tumors^3–14,16^. We sought to address this challenge by detecting cells that express somatic single nucleotide variants (SNVs) in scRNA-seq data.

Detecting genetic variants in scRNA-seq reads is difficult due to the low transcript abundance, allelic dropout, and incomplete transcript coverage inherent to this platform. Despite these challenges, previous studies of intratumoral heterogeneity have demonstrated that single-cell copy number alterations (CNAs) can be robustly detected in full-length cDNAs, commonly generated using the Fluidigm C1/SMART-seq platform, in dozens^3–5,7^ to hundreds^6,8,10,11,14,17,18^ of cells per tumor, and specialized tools have been developed for this purpose^12,19^. Others have built upon plate-based scRNA-seq technologies to detect specific mutations with variable sensitivity^20,21^.

The ability to detect CNAs in single cells has advanced the study of cancers where structural alterations and/or aneuploidy are common^3–10,13,14,18^. However, CNAs are rare in some tumor types, such as AML^22,23^. Moreover, CNAs rarely capture the complete subclonal complexity of any tumor, and are often subclonal progression-associated events^24^. The ability to detect multiple, arbitrary SNVs in scRNA-seq reads is an ideal attribute for any generally-applicable approach to the study of intratumoral heterogeneity. Although previous studies have established that some SNVs can be identified from full-length cDNAs, low numbers of identified mutant cells made downstream analyses difficult^4,5,14^.

In working with the 10x Genomics Chromium Single Cell 3′ (v2) and 5′ (v1) Gene Expression workflows, we observed sequence coverage far from the 3′ and 5′ ends of genes (respectively). This was unexpected, given the end-bias of the Chromium library design, and raised the possibility that the resulting scRNA-seq data could be used for variant detection. Because this platform can sample up to 10,000 cells per library, we hypothesized that even sparse transcript coverage – which would permit the identification of mutations in a fraction of cells – might allow us to combine variant detection with high-throughput transcriptome characterization. Here, we evaluate the utility of 10x scRNA-seq data for somatic variant detection in cryopreserved AML bone marrow samples. Because genome sequencing of paired tumor/normal samples is the gold standard for de novo discovery of somatic mutations and inference of subclonal architecture, we first use “enhanced” whole-genome sequencing (eWGS) of paired tumor/normal samples to discover somatic mutations, and then focus on detecting those mutations in the scRNA-seq data.

## Results

### eWGS and bulk RNA-sequencing

**Fig. 1:**
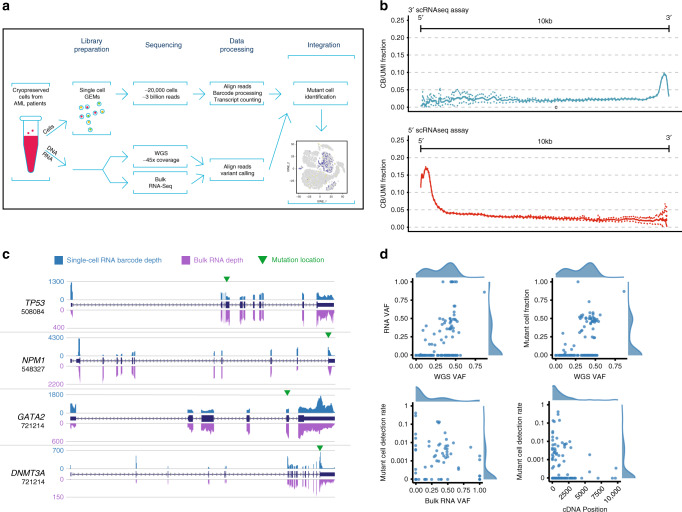
Workflow, coverage, and performance metrics for variant detection in single cells. **a** Cryopreserved bone marrow cells from AML patients underwent eWGS, bulk RNA-seq, and scRNA-seq. Somatic mutations were discovered using eWGS data, identified in individual cells using scRNA-seq data, and interpreted in the context of expression heterogeneity. **b** Fraction of unique transcripts (molecules) whose reads map to any given position up to 10 kbp away from the capture site in both the 5’ and 3’ kits. **c** Comparison of single-cell and bulk RNA-seq coverage data for specific genes of interest. **d** Relationship between RNA and eWGS VAF; dependence of Mutant Cell Fraction on eWGS VAF; dependence of Mutant Cell Detection Rate on bulk RNA VAF, and dependence of Mutant Cell Detection Rate on position of the mutation in the cDNA

Four cases of de novo AML and one of secondary AML were selected for study (clinical details in https://github.com/genome/scrna_mutations). eWGS was used in conjunction with well-established variant detection pipelines to generate a set of high-confidence mutation calls for each case, and bulk RNA-sequencing was used to determine which mutations were expressed in each tumor sample (Methods)^25^. eWGS (Fig. 1a) revealed that these cases were genetically representative of AML, containing on average 26 mutations within coding regions, with many in well-established driver genes (e.g. *DNMT3A*, *FLT3*, *NPM1*, *TP53, NRAS, IDH1, CEBPA*, etc.). To define the clonal architecture of each tumor, the SciClone algorithm^26^ was used to cluster mutations and infer subclones. At least one subclone was identified in every case (Table 1, Supplementary Data 1)^23^. Bulk RNA-sequencing showed that on average, fewer than half of the mutations detected by eWGS were expressed (Table 1).

**Table 1:** Overview of mutation discovery and detection in eWGS and scRNA-seq data

Sample	508084	548327	721214	782328	809653	Mean [SD]
No. cells	14,964	11,620	20,474	21,731	21,038	17,965 [3979]
Reads/cell	192,427	346,965	176,035	214,284	189,751	223,892 [62,745]
Reads mapped confidently to genome (%)	84.9	87.2	92.2	79.8	90	87 [4.29]
Reads mapped confidently to transcriptome (%)	64.2	63.7	73	61.9	68.8	66 [4.04]
Median genes detected per cell	2405	1383	2260	1376	1829	1851 [428]
Total genes detected	22,645	22,503	23,376	25,389	23,102	23,403 [1041]
WGS variants	19	13	41	31	28	26.4
Expressed WGS variants (bulk)	10	5	18	7	8	9.6
scRNA-seq variants	8	7	17	12	8	10.4
Percentage expressed WGS variants discovered in scRNA-seq	80%	140%	94%	170%	100%	117%
Total mutant cells	669	6042	8200	3354	386	3732
Mutant cells per variant	13–453	1–3012	1–3944	1–2619	1–207	3.4–2047
Mutant cells per variant (median)	32	48	30	111	21.5	48.5
Key WGS variants (no. cells with scRNA-seq coverage at variant position)	*IKBKB*^*V616M*^ (150) *FLT3-ITD* (707) *NUP98-NDS1(1)*	*IDH1*^*R132H*^ (118) *NPM1*^*W288fs*^ (5591) *SRSF2*^*P95H*^ (2349)	*DNMT3A* ^*R882H*^ (409) *FLT3-ITD* (479) *FLT3*^*F612L*^ (306) *NPM1*^*W288fs*^ (11,672) *GATA2*^*R361C*^ (1629)	*NRAS*^*G12S*^ (949) *NRAS*^*G12D*^ (951) *U2AF1*^*S34F*^ (4509)	*NRAS*^*G12D*^ (1412) *TP53*^*E286G*^ (239) *CEBPA* ^*R142fs*^ (84)	N/A
Additional variants with expression signature (number of cells with coverage)	*RNF10 (103)*			*NAGLU* ^*E634K*^ *(216)*		N/A

### Single-cell transcript coverage and representation

We first compared genome-wide transcript coverage obtained from the 5′ (v1) and 3′ (v2) 10x Genomics Chromium Single Cell Gene Expression workflows. For one case, UPN 508084, we generated two scRNA-seq libraries with each workflow, and sequenced them to high depth, targeting 200,000 reads/cell. Transcriptome-wide coverage for each data set was assessed using 20,090 genes, each having one annotated isoform (Methods). Both workflows yielded consistent low-level coverage at least 10 kbp from the 5′ and 3′ ends of the average transcript (Fig. 1b). However, the 5′ kit yielded slightly higher transcript-wide coverage in distal regions of transcripts. For the average gene assayed using that kit, at least 2.5% of the unique sequenced transcripts mapped to any given base up to 10 kbp away from the 5′ transcription start site of the gene. Coverage metrics for 200 cancer-relevant genes are summarized in Supplementary Data 2 and provided at nucleotide resolution at https://github.com/genome/scrna_mutations. Subsequent sequencing and analyses were performed using only the 5′ workflow application.

We then asked whether bulk and single-cell RNA-seq data capture the same transcript structure for the mutated genes in this study. Using one canonical isoform for each gene, we compared coverage in the single-cell data (unique barcode/UMI pairs at each position) to that in the bulk RNA-seq data (quantified using bamCoverage and 1 bp bins), and visualized it using the UCSC Genome Browser (Methods). The results demonstrate that, for each gene studied, bulk- and single-cell data identified the same set of transcripts (Fig. 1c). Coverage plots for all mutated genes in this study are provided at https://github.com/genome/scrna_mutations.

### Mutation identification in single cells

We next sought to identify cells containing any of the somatic variants discovered using eWGS. For each cell and each variant position in the eWGS data, unique wild-type, and mutant reads were counted using cb_sniffer, a tool that extends the PySam library to do barcode-aware pileups (see Methods: https://github.com/genome/cb_sniffer). In most high-throughput scRNA-seq datasets, the median gene is represented in the median cell by one transcript read. Consistent with this, most SNV locations were covered by a single read in most cells (although SNVs in several highly expressed, high-coverage genes (e.g. *U2AF1*, *NPM1*, *SRSF2*, and *NRAS*) were more likely to have multiple reads per cell) (Supplementary Fig. 1a). For a heterozygous mutation, therefore, there is a 50% chance that the observed transcript is mutant, and a 50% chance that it is wild-type, leading to the phenomenon known as allelic dropout. This has two main consequences: first, it is impossible to conclude that a cell is wild-type; secondly, the sensitivity of mutation detection is reduced by a factor of two. Therefore, barring sequencing errors, one can in principle classify a cell’s genotype as “mutant” if it contains one or more mutant transcripts, and “unknown” if it does not. We measured the frequency of false positives originating from sequencing errors by examining the positions of known somatic mutations in samples that did not harbor those mutations. The false-positive rate (the rate at which wild-type UMIs are called mutant in the control samples) was site-specific, and had a maximum rate of only 0.39% (Supplementary Data 3). We also searched for these variants in 8057 bone marrow cells from four healthy donors, and found no false positives.

We therefore labeled a cell “mutant” if it contained at least one variant-containing read, and “unknown” if only wild-type reads or no reads were detected. We found an average of 49 mutant cells per variant (range: 1–3944), and 3732 mutant cells (22% of the total cells) per sample, but this varied widely among samples (range: 396–8200, or 1.8–52%), depending on the mutations present in each (Table 1). Most mutant cells contained one detected mutation, with one read mapping to the variant position (Supplementary Fig. 1). Founding clone mutations, subclonal mutations, and putative driver mutations were detectable in each case, and these included SNVs, insertions and deletions (indels, including *FLT3*-ITD and *NPMc*), and one gene fusion (*NUP98-NSD1*) (Table 1, Supplementary Data 1). Although the vast majority of mutant cells contained only one mutation (88–98%, depending on the sample), a small fraction of cells in each case contained multiple mutations, particularly when a founding clone mutation was readily detectable (Table 2). Specifically, two mutations were found in 1.6–12% of the mutant cells in a sample; three mutations were found in 0.21–0.29% of mutant cells; and four mutations were found in one cell (0.012%) in sample 721214. The observed mutation combinations were consistent with the known subclonal architecture (although the mutation data was generally not dense enough for accurate de novo subclonal inference). For example, case 548327 contained an *NPM1*^*W288fs*^ mutation in the founding clone, and several hundred cells contained both this mutation and one subclonal mutation. Case 721214 is composed of three subclones sequentially nested within the founding clone. One cell was found to have one mutation from each (sub)clone.

**Table 2:** Frequency of cells containing multiple mutations in each case

Sample	508084	548327	721214	782328	809653
Total mutant cells	669	6042	8200	3354	396
1 mutation (%)	658 (98)	5290 (88)	7702 (94)	3176 (95)	386 (97)
2 mutations (%)	11 (1.6)	734 (12)	477 (5.8)	171 (5.1)	10 (2.5)
3 mutations (%)	0	18 (0.29)	20 (0.24)	7 (0.21)	0
4 mutations (%)	0	0	1 (0.012)	0	0

Mutation detection in single cells was compared to that in bulk RNA-seq and eWGS data using a read-based metric, “single-cell Variant Allele Frequency” (scVAF), which enabled us to compare VAFs across data types, and two cell-based metrics, “Mutant Cell Fraction” (MCF) and “Mutant Cell Detection Rate” (MCDR), which allowed us to measure and compare the sensitivity of mutant cell identification (Methods). In terms of mutant reads, the sensitivity of mutation detection was comparable in single cell and bulk RNA-seq data: on average, a slightly higher fraction of known mutations was detected in the scRNA-seq data, but not necessarily in a large number of cells (Table 1). We then examined the relationship between bulk VAF (either eWGS or bulk RNA-seq) and single-cell VAF (scVAF) and detection sensitivity (MCF): for expressed mutations that were identifiable in bulk and single-cell RNA-seq data, single-cell MCFs and scVAFs were more highly correlated with eWGS VAFs (*r* = 0.69 and 0.68, respectively) than were bulk RNA VAFs (*r* = 0.52; Table 1). scVAFs were positively correlated with bulk RNA-seq VAFs (*r* = 0.34; Fig. 1d, Supplementary Fig. 1c). Although mutation-detection in scRNA-seq is sensitive from a read-based perspective, sensitivity from a cell-based perspective is very low and mutation-specific; we identified only a small fraction of the cells that would be expected to contain mutations based on eWGS VAFs (MCDR, Fig. 1d). This fraction depends on multiple variables, including bulk RNA VAF, distance from the 5′ end of the transcript, and single-cell gene expression (Fig. 1d, Supplementary Fig. 1c, d).

The ability to detect mutations in scRNA-seq data therefore depends on a number of variables, including VAF, expression level of the mutated gene, position of the mutation in the transcript, sequencing depth, fraction of tumor cells in the sample, and number of cells sequenced. The probability of finding at least one cell containing a particular heterozygous mutation *m* is approximately:

1 $$P\left( m \right) = na\left( {f\left[ {1 - \left( {1 - ct} \right)^r} \right] + \left( {1 - f} \right)e} \right) \approx nafrct + na\left( {1 - f} \right)e$$

Where *f* is twice the variant allele frequency of the mutation in the eWGS data, *t* is the relative expression level of the gene (e.g. in counts per million), *r* is the average number of UMIs per mutant cell, *c* is the fraction of UMIs that have coverage at the mutant position, *e* is the site-specific false-positive rate (frequency with which a wild-type cell is called mutant), *a* is the fraction of cells in the sample that are tumor cells, and *n* is the total number of cells sequenced.

### Using SNVs to distinguish between tumor and normal cells

Single-cell CNA detection is often used to identify tumor cells in samples that contain a mixture of tumor and normal cells, but sensitivity is limited by the fact that CNAs are frequently subclonal, even in the (non-AML) tumors that contain them^24^. Therefore, we investigated the utility of single-cell SNV detection for this purpose. A straightforward approach would involve selecting only those cells that contain a mutation; we detected an average of 3732 mutant cells per sample (Table 1). Despite the wide range (396–8200), this is substantially more than the total number of cells/sample analyzed in previous single-cell mutation-detection studies^3–10,13,14^. However, we retained the additional cells in each sample (which contained valuable expression information), and instead used single-cell SNVs as markers for tumor vs. wild-type cell clusters.

**Fig. 2:**
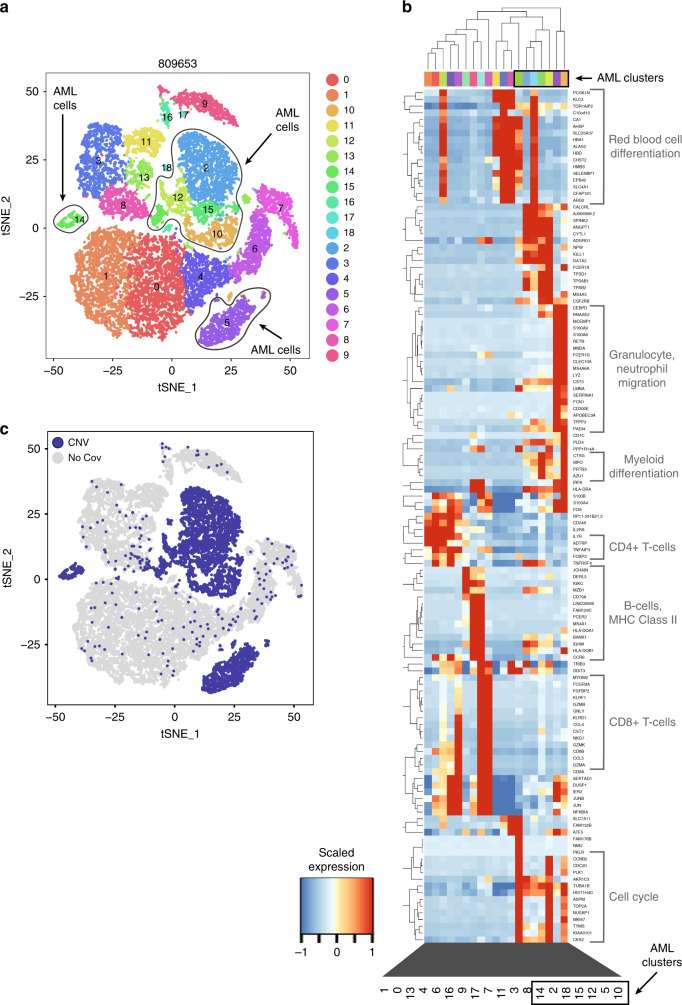
Clustering, overview of expression heterogeneity, and copy number analysis in 809653. **a** t-SNE projection of scRNA-seq data, with cells colored according to graph-based cluster assignment; putative AML clusters (based on later analyses) circled. **b** Hierarchical clustering of the most heavily weighted genes in each principal component, averaged within graph-based clusters. Each column represents a cluster from panel **a**. **c** CNV analysis: blue, cells with detected CNVs; gray, no detected CNVs

We first used principal component analysis to summarize the expression heterogeneity in each case (Methods) to better understand the composition of each sample. As expected, this revealed complex relationships among clusters (such as partially overlapping expression signatures), and multiple sources of heterogeneity in all samples, including variable expression of known hematopoietic cell-type markers (e.g. *CD3D* (T-cells), *CD79A*, or *CD19* (B-cells), and *HBA1* (erythrocytes)), cell cycle genes (e.g. *TUBA1B*, *TOP2A*), markers of myeloid lineage (e.g. *AZU1*, *ELANE*, *MPO*, *PRTN3*), mitochondrial genes, and ribosomal genes (Fig. 2a, b; Supplementary Fig. 2–5, Supplementary Data 4). This indicated that the distribution of cell types is a major source of expression heterogeneity, and varies among samples, as expected.

**Fig. 3:**
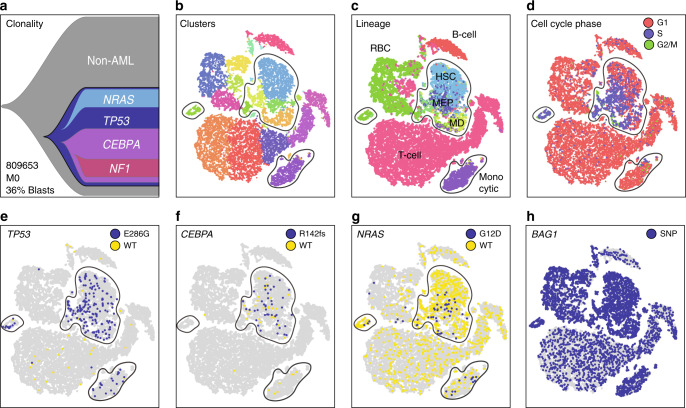
Single-cell mutation detection and interpretation in case 809653. **a** Clonality inferred from eWGS, with subclonal driver genes labeled. **b** t-SNE projection of scRNA-seq data with cells colored according to graph-based cluster assignment. In panels **b**–**g**, putative clusters of AML cells are circled. **c** Cells colored according to inferred lineage; RBC = red blood cell, HSC = hematopoietic stem cell, MEP = myeloid-erythroid progenitor, MD = myeloid dendritic cell. **d** Cells colored according to cell cycle phase. **e**–**g** Cells colored according to single-cell genotype at the *TP53*^E286G^, *CEBPA*^R142fs^, and *NRAS*^G12D^ sites: blue, at least one mutant read detected; yellow, wild-type reads only; gray, no coverage. **h** Cells colored according to single-cell genotype at the homozygous *BAG1* germline SNP: blue, at least one mutant read detected; gray, no coverage

**Fig. 4:**
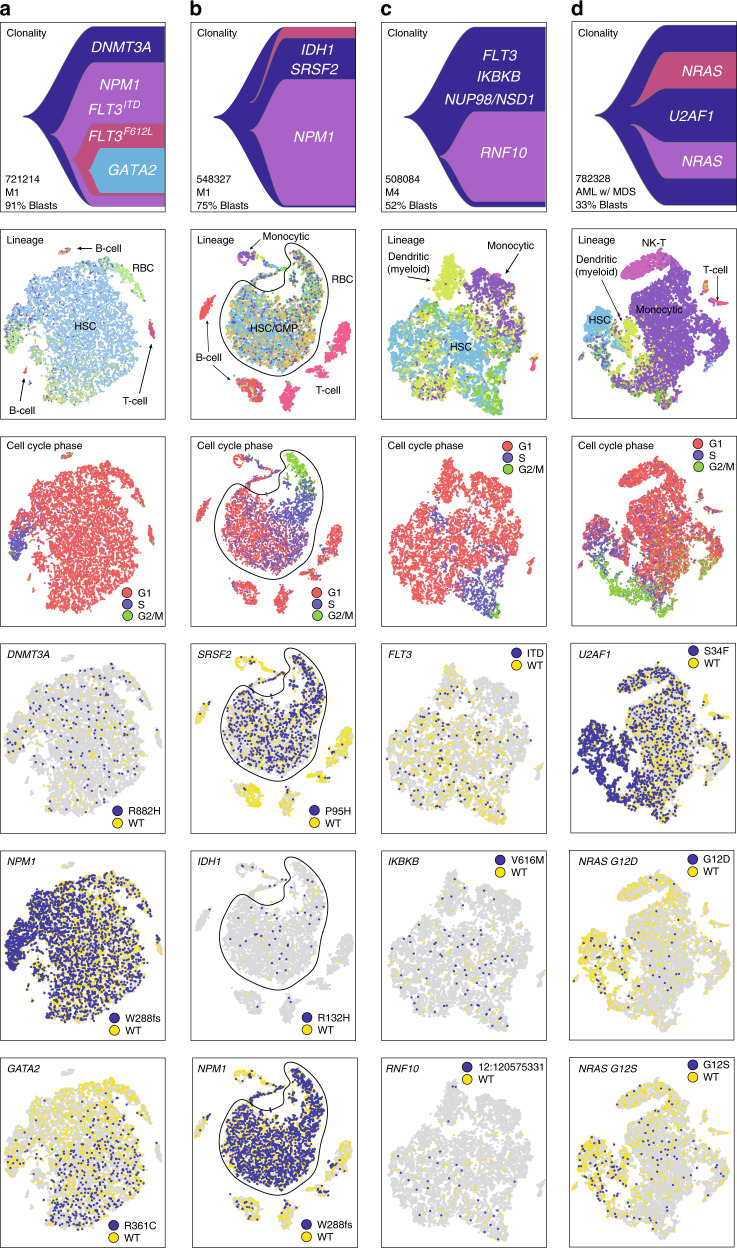
Single-cell mutation detection and interpretation in additional cases ordered by the differentiation signature of AML cells. **a** 721214, top to bottom: clonality inferred from eWGS; cells colored according to closest inferred lineage (RBC = red blood cell, HSC = hematopoietic stem cell, CMP = common myeloid progenitor); cells colored according to cell cycle phase; cells colored according to single-cell genotype at the indicated site: blue, at least one mutant read detected; yellow, wild-type reads only; gray, no coverage. **b** 548327, putative AML cells circled. **c** 508084. **d** 782328

To investigate sample composition in a more unsupervised manner, we identified the nearest hematopoietic lineage of each cell by matching each cell’s expression profile to the most similar lineage-specific expression profile in the DMAP database^27^ (Methods, Figs. 3c and 4). The inferred sample composition varied widely among subjects, particularly with respect to the fraction of lineage-defined cells (e.g. cells resembling myelomonocytic cells, T-cells, B-cells, and erythrocytes). All five samples contained clusters of immature cells, including cells resembling hematopoietic stem cells (HSCs), common myeloid progenitors (CMPs), and megakaryocyte-erythroid progenitors (MEPs), which could represent either immature non-malignant cells or AML cells.

To clarify the identity of these clusters, we combined single-cell mutation detection with expression-based clustering and lineage inference. Using the bone marrow sample from 809653 (which contained many non-AML cells, based on morphology and flow cytometry) we overlaid mutation data on the t-SNE projections by highlighting mutant cells (Fig. 3e–g). A highly expressed germline SNP in the *BAG1* gene served as a positive control, marking SNP-containing cells in all expression clusters (Fig. 3h). By scRNA-seq, we detected cells expressing mutations in 8 genes, including *TP53*, *NRAS*, and *CEBPA* (Table 1, Supplementary Data 1). Several clusters were significantly enriched (*p* ≤ 0.05, one-sided Fisher exact test) for mutant cells; other cells in these clusters presumably contained undetected mutations in these genes (Fig. 3b–g). Two of these clusters were composed of cells that had stem/progenitor expression signatures (HSCs and MEPs). The other two were composed of cells expressing erythrocyte or monocyte markers; in terms of gene expression, these clusters are distinct from normal cell clusters, but they could not have been labeled as AML-derived using expression data alone.

This was the only case with multiple CNAs, allowing us to benchmark SNV-based cell classification against the better-established CNA-based methods. CONICSmat^19^ was used to identify cells containing the CNAs discovered by eWGS, and high concordance was observed with SNV/expression-based classification of AML cells: 95.5% of cells classified as AML by copy number were also classified as AML by SNV and expression signature (Fig. 2c). Conversely, 94.9% of cells classified as AML by SNVs and expression were confirmed by CNA analysis. This demonstrated two key points: first, SNV-based classification of AML clusters can perform comparably to CNA-based methods, and second, cells in mutation-enriched clusters are also likely to be AML cells, even if they contain no detectable mutation.

In the other 4 cases, somatic mutations were also concentrated in specific cell clusters, suggesting that they represented AML cells (Fig. 4). This approach to AML cell identification, which assumes that all cells in mutation-enriched clusters are likely to be AML cells, may miss small clusters of mutant cells, and rare AML cells that co-cluster with cells of different lineages; an alternative approach is to analyze only (and all) cells with identified mutations (below). Overall, combining expression and mutation data delineated clusters of AML cells more comprehensively than either method alone, and allowed us to identify abnormally-differentiated AML cells (“lineage infidelity”^28^).

### Evaluating tumor differentiation state

By combining lineage inference with single-cell mutation identification, we estimated the extent of differentiation of each tumor. Our conclusions were supported by flow cytometry and morphology, but provided more insight into the differentiation state of AML cells in individual samples (Figs. 3 and 4). In two cases (809653 and 782328), a considerable fraction of the mutant cells had expression signatures consistent with differentiated cells: erythrocytes and monocytes in 809653 (Fig. 3c), and monocytes and NK-T cells in 782328 (Fig. 4d). Likewise, case 548327 contained mutant cells that co-clustered with wild-type B- and T-cells, again suggesting that some AML cells display lineage infidelity (Fig. 4b). Thus, this integrative genomic approach validates the concept that AML cells can have a variety of abnormal expression signatures, corresponding to different lineages and states of differentiation.

### Expression signatures of mutation-containing cells

In the approach described above, we treated mutation-containing cells as markers for entire clusters of putative AML cells. However, the ability to map mutations in many cells (3732 mutant cells/sample on average) facilitates more conservative, direct analyses of intratumoral expression heterogeneity, using only the cells that express a confirmed somatic mutation. For each sample, therefore, we analyzed the mutationally defined AML cells separately (Methods, Supplementary Figs. 6 and 7). As expected, all samples showed intercellular heterogeneity in the expression of cell cycle genes (as expected) and genes that function in the immune system, especially the MHC Class II genes and/or *CD74*. All but one case (782328) showed intercellular variability in expression of *TP53*-interacting genes^29^. Three cases (508084, 548327, and 721214) showed intercellular heterogeneity in genes that interact with the vascular cell adhesion gene *VCAM1*, and three (721214, 782328, and 809653) showed heterogeneous expression of myeloid differentiation genes. There were also case-specific signatures, such as “response to reactive oxygen species” in 721214^29^. As discussed further below, a *GATA2*^R361C^ expression signature is evident in cells expressing this mutation. Thus, the reduced, mutant- only data set is sufficient to capture much of the expression heterogeneity observed in the total sample.

### Mutation-associated expression signatures

We next investigated the extent to which mutational heterogeneity was associated with transcriptional heterogeneity in each case. A subclonal mutation that drives an expression signature should be restricted in expression space. In contrast, a founding or subclonal mutation not associated with an expression signature should be present throughout expression space. Furthermore, this should not depend on restricted expression of the mutant gene. To this end, we highlighted mutant cells on the t-SNE projection of each sample, and identified mutations that are nonuniformly distributed, even after controlling for that gene’s expression (Figs. 3e–g and 4, Supplementary Fig. 8). We performed two versions of this analysis: a “whole-sample” analysis using all cells, and a “mutant-cell” analysis using only mutation-containing cells. The whole-sample analysis capitalized on the high throughput of this platform by incorporating expression information from all ~20,000 cells per sample, thereby improving our ability to discern distinct expression signatures. On the other hand, the limitations of genotype assignment imposed by low coverage and allelic dropout required that we compare a relatively small number of mutant cells to a much larger number of cells of unknown genotype (representing a mixture of mutant and wild-type cells). Therefore, instead of performing a straightforward comparison between mutant and wild-type cells, we looked for evidence that the mutant cells were nonuniformly distributed among the “unknown” cells: when defining expression signatures, as described further below, we searched for genes whose expression was correlated with the density of mutant cells.

The results of the whole-sample analysis indicated that the relationship between expression heterogeneity and mutational heterogeneity is case- and mutation-dependent. Two cases, 721214 and 508084, contained subclonal mutations with nonuniform distributions (Fig. 4a, c). Based on eWGS, 721214 contained a subclone defined by *GATA2*^R361C^. In the scRNA-seq data, cells expressing *GATA2*^R361C^ were largely restricted to the same space on one side of the t-SNE projection, suggesting that AML cells containing this mutation have a unique expression signature (Fig. 4a). Two cases (809653 (Fig. 3f–g) and 782328 (Fig. 4d)) exhibited complex mutation-associated expression profiles, and a third, 548327 (Fig. 4b), showed expression heterogeneity in the absence of discernable genetic heterogeneity. The *GATA2*^R361C^ gradient in 721214 was of particular interest, because *GATA2* encodes a transcription factor that is a key regulator of hematopoiesis, and is recurrently mutated in AML^23,30^. We therefore sought to characterize the associated expression signature.

**Fig. 5:**
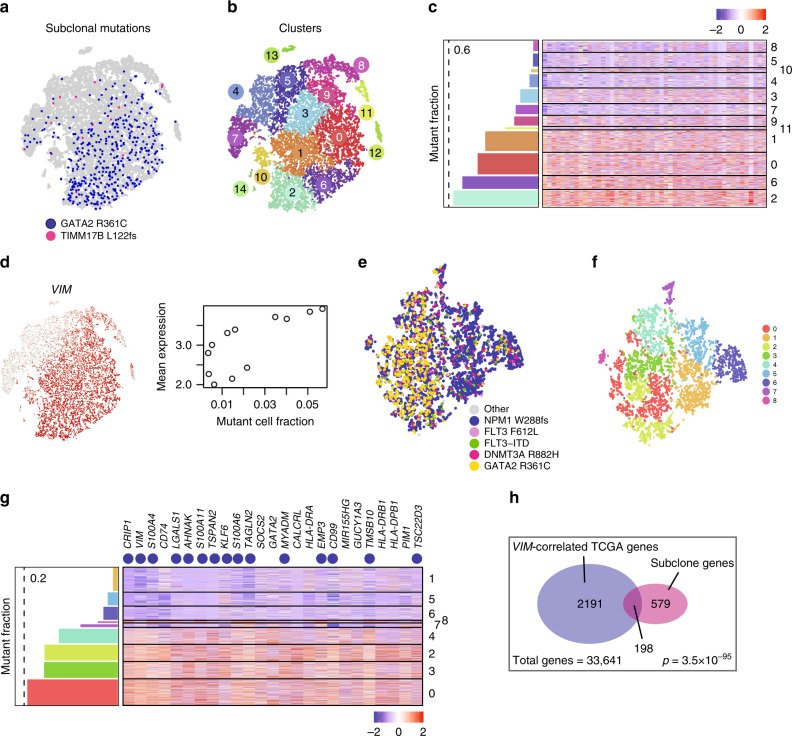
*GATA2*^R361C^ Subclonal expression signature. **a** t-SNE projection showing mutation-expressing cells in blue (*GATA2*^R361C^) and pink (*TIMM17B*^L122fs^). **b** Cells colored according to graph-based cluster assignment. **c** Heatmap of top 50 mutation-dependent genes, with bar graph showing mutant cell fraction in each AML cluster (labeled to the right of the heatmap). **d** Cells colored according to *VIM* expression (left), and scatterplot showing average *VIM* expression in each cluster as a function of the subclonal mutation fraction of each cluster (right). **e** t-SNE plot constructed from mutant cells, which are colored according to the mutation they contain: *GATA2*^R361C^, yellow; *DNMT3A*^R882H^, pink; *FLT3*-ITD, green; *FLT3*^F612L^, purple; *NPM1*^W288FS^; other somatic mutation(s), gray. **f** Mutant cells colored according to graph-based cluster. **g** Heatmap of top 25 subclonal mutation-dependent genes, with bar graph showing mutant cell fraction in each cluster (labeled to the right of the heatmap). Genes that are highly correlated with *VIM* in TCGA are indicated with blue dots. **h** Venn diagram indicating gene sets used to identify the *VIM* regulome

As noted above, scRNA-seq data allows us to distinguish between mutant cells and cells of unknown genotype; we cannot conclusively label a cell as “wild-type.” To address this limitation while incorporating expression information from cells of unknown genotype, we employed a regression-based method that identifies genes whose expression is correlated with the density of mutant cells (Methods), for a given mutation or set of subclonal mutations. This approach makes use of expression clusters to smooth the expression and density data, but does not depend on the exact clustering, and does not require us to identify cluster-specific gene expression. It also permitted us to control for potential covariates, such as the expression of *GATA2*, which was slightly correlated with the mutation density gradient. We applied this method to the full data set in two ways: first, by searching for genes whose expression was correlated with any mutation in the *GATA2*^R361C^ subclone (Fig. 5a), and second, for genes associated with *GATA2*^R361C^ per se. Each analysis yielded several hundred genes whose expression was positively correlated with *GATA2*^R361C^ density (FDR-adjusted *p-*value for the regression coefficient <0.05; Fig. 5b, c, Supplementary Data 5a, b). Clusters with a higher density of expressed *GATA2*^R361C^ subclonal mutations exhibited higher expression of genes involved in immune response, apoptosis, and leukocyte adhesion^29^ (Fig. 5c, Supplementary Table 1). Notably, the gene whose expression is most strikingly correlated with this subclone is *VIM*, which encodes a type III intermediate filament and is an established target of the *GATA2*/*SPI1* (PU.1) transcriptional circuit (Fig. 5d)^31,32^.

As described above, we also analyzed this sample using only the cells of known (i.e. mutant) genotype, and again found that *GATA2*^R361C^ subclonal mutations were nonuniformly distributed in expression space. We compared mutant-rich clusters (mutation fraction >10%) to the remaining clusters using a Wilcoxon rank sum test for differential expression^33^ (Fig. 5e–h). Consistent with the above results, subclonal mutations were associated with higher expression of *VIM*, *CRIP1*, *AHNAK*, *CD74*, and other genes associated with immune response, apoptosis, and cell adhesion (Supplementary Data 5c).

Many of the *GATA2*^R361C^ subclone-associated genes are highly correlated with each other (and with *VIM)*, in the TCGA AML gene expression data^34^. To quantify the overlap between TCGA *VIM*-associated genes and *GATA2*^R361C^ subclone-associated genes, we identified 2191 genes that are highly correlated with *VIM* in TCGA (*q* < 0.001, Pearson correlation, Benjamini-Hochberg correction), and used a hypergeometric test to compare them to the *GATA2*^R361C^ subclone-associated genes (Fig. 5h). The intersection of these gene sets was statistically significant (*p* = 3.5 × 10^–95^, hypergeometric test), suggesting the existence of a *VIM* “regulon” whose expression is influenced by one or more mutations in the *GATA2*^R361C^ subclone. To further characterize this regulon, we examined the functional enrichment of the 198 genes in the intersection (Supplementary Table 2, Supplementary Data 5d), and found that they are enriched for Gene Ontology (GO) terms related to immune response (in particular, the Fc-gamma receptor pathway), cytoskeletal organization, and focal adhesion, and for genes that interact with *WAS*. *WAS*, which encodes Wiscott-Aldrich Syndrome Protein, transduces signals from the cell surface to the actin cytoskeleton in response to infection, and is required for a variety of immunological cell functions. *WAS* mutations are associated with a broad spectrum of clinical manifestations, including immunodeficiencies and hematologic malignancies^35^. Like *VIM*, *WAS* may also be regulated by PU.1^36^. Together with our data, this suggests that a subset of PU.1 target genes coordinates immune function with cytoskeletal reorganization in hematopoietic cells, and that at least one of the mutations in the *GATA2*^R361C^ subclone influences the expression of these genes. Because *GATA2* is a transcription factor that negatively regulates PU.1^32^, it is likely that the *GATA2*^R361C^ mutation itself is at least partly responsible for the observed transcriptional effects in this sample. Furthermore, *GATA2* has well-documented roles in both immune function and hematological malignancies: autosomal dominant mutations in *GATA2* can also lead to immunological disorders and hematologic malignancies^37,38^.

**Fig. 6:**
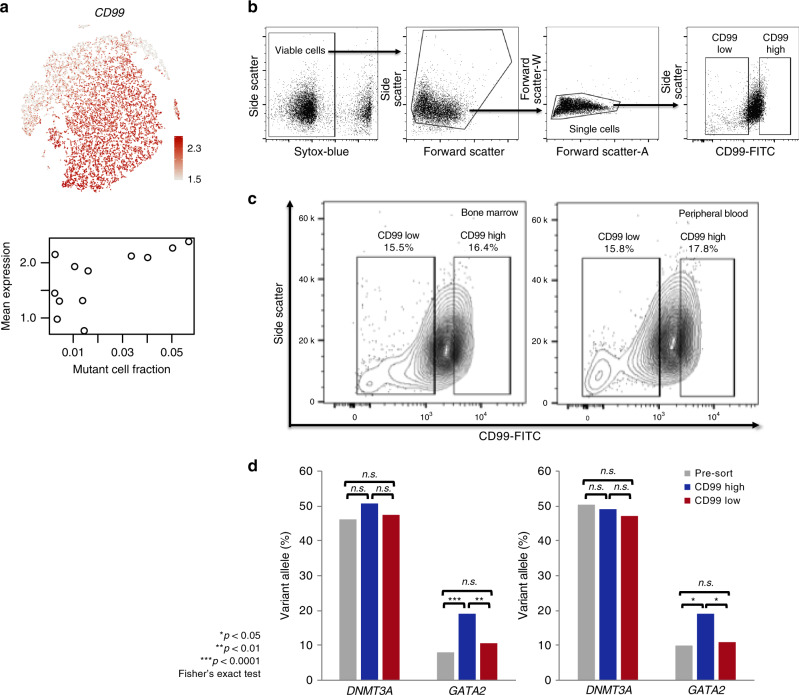
Orthogonal confirmation of *GATA2*^R361C^ expression signature by flow cytometry and targeted sequencing. **a** Cells colored according to *CD99* expression (top), and scatterplot showing average *CD99* expression in each cluster as a function of the *GATA2* mutation fraction of each cluster (bottom). **b** Sorting strategy: Cells staining positive for the dead cell dye Sytox-blue were excluded, then debris was excluded. Singlets were gated for final sorting of CD99-low and CD99-high expressing populations. **c** Gating of cells based on *CD99* expression using flow cytometry (bone marrow, left; peripheral blood, right). **d** Variant allele fraction of the founding clone *DNMT3A*^R882H^ mutation and the subclonal *GATA2*^R361C^ mutation in unsorted cells (gray), CD99-high cells (blue), and CD99-low cells (red) (bone marrow, left; peripheral blood, right)

The success of this method depends on a number of factors, including steepness of the expression gradient and number of mutant cells (the more subtle the expression signature, the more mutant cells required). Moreover, irrelevant or hidden variables can affect the distribution of mutant cells in expression space, such as expression level of the mutated gene, cell cycle phase, ribosomal transcript content, mitochondrial transcript content, or other variables for which we could not account. We therefore used an independent experimental approach to test for the *GATA2*^R361C^-associated expression gradient, in which we compared the frequency of *GATA2*^R361C^ in genomic DNA from cells drawn from each extreme of the expression gradient. First, scRNA-seq was used to identify cell-surface markers whose expression was correlated with the *GATA2*^R361C^ mutation gradient. This analysis yielded CD99, a well-described cell-surface marker associated with AML and MDS cells^39^ (Fig. 6a). Then, peripheral blood and bone marrow samples from this patient were stained with CD99 FITC-conjugated antibody, and flow cytometry was used to isolate cells with high or low CD99 expression (top or bottom 15%, Fig. 6b, c; Methods). Genomic DNA was prepared from each population, and targeted sequencing was used to measure the frequency of *GATA2*^R361^ mutations (as well as a control *DNMT3A*^R882^ mutation, which is found in all AML cells in this sample) in each cell population. This demonstrated that *GATA2*^R361C^ is significantly more abundant (*p* = 0.0081 (marrow), *p* = 0.0432 (peripheral blood), Fisher Exact test) in the genomes of the CD99^hi^ cells (Fig. 6d). The control mutation, *DNMT3A*^R882^, was not significantly enriched in the CD99^hi^ cells, because it is present in all of the AML cells in this sample (it was the initiating event)*. GATA2*^R361C^ abundance varies more dramatically in the scRNA-seq data, possibly due to allele-specific expression of the mutant allele, a documented phenomenon in *GATA2*-mutated AML^40^. These results support the conclusion that *GATA2*^R361C^ is associated with a distinct gene expression profile, and shows that SNV detection in scRNA-seq data can be used to identify mutation-associated expression signatures. Moreover, the ability to identify cell surface markers for the purification and analysis of subclones is an important application of scRNA-seq data that should have broad applications.

**Fig. 7:**
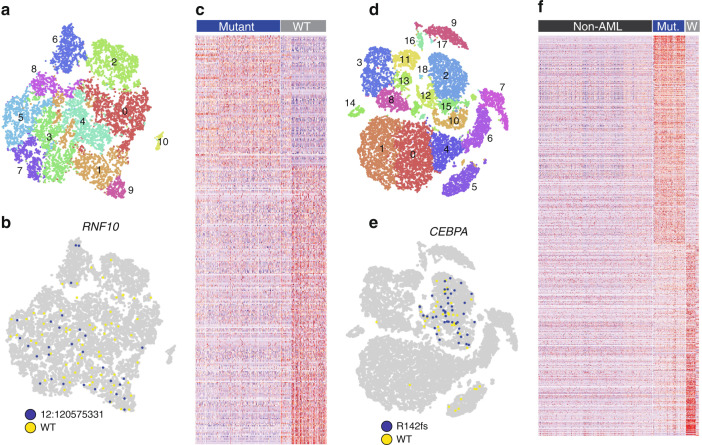
Additional mutation-associated gene expression signatures. **a** t-SNE plot of 508084, cells colored by graph-based cluster. **b** Cells colored according to coverage at the *RNF10*^NULL^ site (mutant = blue, wild-type = yellow). **c** Genes that are differentially expressed between mutant-rich and mutant-poor clusters. **d** t-SNE plot of 809653, cells colored by graph-based cluster. **e** Cells colored according to coverage at the *CEBPA*^R142fs^ site (mutant = blue, wild-type = yellow). **f** Genes that are differentially expressed between mutant-rich and mutant-poor clusters; non-AML cells shown for comparison

In case 508084, a mutation in *RNF10*, a putative transcription factor of unknown function, was also restricted to a subset of expression clusters (Fig. 7a, b). Compared to clusters with few or no mutant cells, mutant-rich clusters displayed a clear expression signature marked by high expression of genes involved in immune-related cell adhesion. This included genes involved in MHC Class II receptor activity and T cell aggregation, as well as genes that interact with MCM2 (a regulator of TP53) and NPM (which is frequently mutated in AML) (Fig. 7c, Supplementary Table 3, Supplementary Data 5e).

The remaining three cases showed more complex relationships between expression and mutations. Based on the eWGS results, 809653 contained *TP53*^E286G^ and *CEBPA*^R142fs^ in the founding clone, and a subclone defined by *NRAS*^G12D^ and *NF1*^I679fs^. In the single-cell data, however, the distribution of *CEBPA*^R142fs^ was markedly nonuniform, suggesting that *CEBPA*^R142fs^ may be in a subclone (Fig. 7d, e); the clonal architecture of this case may be more complicated than can be discerned from a single eWGS sample. Notably, cells expressing *CEBPA*^R142fs^ were restricted to one AML cluster that differed from the other AML clusters with respect to differentiation state and cell cycle status: compared to *CEBPA* wild-type AML clusters, the *CEBPA*-mutant cluster was enriched for cells in S-phase, and cells with progenitor-like expression signatures. Differential expression analysis of the *CEBPA*-mutant cluster showed that it overexpressed genes associated with a variety of biological processes, most notably ribosome biogenesis, which probably reflects the increased protein synthesis requirements of rapidly proliferating cells (Fig. 7f, Supplementary Table 4, Supplementary Data 5f). The cluster also expressed a variety of key transcription factors involved in myeloid differentiation, particularly targets of Myc, consistent with the observed perturbation of cellular differentiation in this cluster.

In 782328, *NRAS*^G12D^ and *NRAS*^G12S^ also display subtle expression signatures (Fig. 4d). They are predominantly localized to cells in the S, G2, and M phases of the cell cycle, suggesting a role for these mutations in cell cycle progression or proliferation rate. Further work will be required to characterize and confirm these putative mutation-expression associations.

### Interaction between genetic and epigenetic heterogeneity

The interplay between genetic and epigenetic heterogeneity could have important consequences for intratumoral phenotypic heterogeneity: a mutation will likely have functional effects only in the cells in which it is expressed. This phenomenon would manifest in scRNA-seq data as a mutation that is confined to a portion of expression space by virtue of the fact that the mutation-containing gene is only expressed in that region; the converse is not necessarily true, due to the potential for dropouts in scRNA-seq data. In case 508084, we observed a subtle mutation gradient for the *FLT3-ITD* mutation in case 508084, which was caused by a corresponding *FLT3* expression gradient. This phenomenon was also observed in the *CEBPA*^R142fs^ mutation gradient in 809653, which was partly due to heterogeneous *CEBPA* expression.

## Discussion

The ability to link genetic and transcriptomic information in single cells has important implications for the study of heterogeneous cell populations. By combining eWGS and scRNA-seq data from a high-throughput platform, we can distinguish between tumor and non-tumor cells, identify tumor cells displaying lineage infidelity, evaluate the differentiation state of individual tumor samples, derive mutation-associated expression signatures, study transcriptional heterogeneity within confirmed tumor cells, and identify cell-surface markers that can be used to isolate specific cells for downstream studies. Further, the approach described here should be applicable–without additional modifications or customization–to virtually any tumor type.

Previous studies have demonstrated that CNAs and specific genetic variants, such as the *BCR-ABL* fusion, can be identified with high sensitivity in full-length transcripts from dozens to hundreds of single cells using plate-based techniques such as the Fluidigm C1/Smartseq platform, and that SNVs can also be identified, albeit with lower sensitivity, using that data. Because CNAs rarely reflect the complete clonal architecture of a tumor (and are rare in most AML samples), we were interested in finding a way to identify SNVs in single cells. We noticed that the 10x Genomics Chromium Single Cell 3′ and 5′ Gene Expression workflows yield unexpectedly high transcript coverage far from the 3′ and 5′ ends of transcripts. Although this distal coverage is sparse, it is sufficient for low-sensitivity variant detection in single cells: SNVs were detectable in 22.7% of the cells in our samples, on average. Coupled with the high throughput of the platform, this sensitivity enables the detection of SNVs in hundreds to thousands of cells per sample. Although these cells can be studied in isolation, we analyzed them in the context of the entire sample, thereby leveraging the expression information provided by the additional, non-genotyped cells.

A common application of variant detection in scRNA-seq data is to distinguish tumor from normal cells in heterogenous samples. However, because malignant cells can have expression profiles that mimic more highly differentiated normal cells, gene expression data alone is not sufficient to identify bona fide AML cells. Moreover, AML cells sometimes display lineage infidelity, where some AML cells display the characteristics of differentiated cell types from other lineages, such as T-cells. These AML cells, which would have been missed if classification had been performed using expression signatures alone, can be identified when mutation information is also considered.

Transcriptional heterogeneity in AML samples clearly arises from multiple sources, including the differentiation states of normal and tumor cells, cell cycle states, mutations that are present in subsets of cells (i.e. subclones), or non-genetic heterogeneity that arises as a consequence of stochastic gene expression or other perturbations. Incorporating variant detection into scRNA-seq analysis helps to distinguish among these sources by facilitating the distinction between tumor and normal cells, and by revealing correlations between mutational and transcriptional heterogeneity. Equally, importantly, it suggests that genetic heterogeneity plays a limited role in establishing transcriptional heterogeneity: we routinely observed expression heterogeneity in the absence of detectable genetic heterogeneity. In some cases, this may be due in part to the limited sensitivity of mutation detection. In others, it may exemplify the well-established phenomenon whereby stochastic gene expression gives rise to phenotypic heterogeneity in clonal populations of cells^41–43^. Non-genetic transcriptional heterogeneity can influence phenotype (such as drug sensitivity^44,45^, growth rate^46^, and cell fate^43^) and persist across generations^46–48^, and might therefore serve as a substrate for natural selection^49^. This underscores the idea that a combination of genetic and non-genetic sources of heterogeneity may help to govern tumor biology and evolution. As additional scRNA-seq studies of primary tumor samples are undertaken by many groups, the relative contributions of these sources of heterogeneity for each tumor type should become more clear.

The detection of cells with expressed mutations in scRNA-seq data is subject to several limitations. Dropout (including transcript dropout and allelic dropout) occurs with most scRNA-seq platforms. As a result, it is impossible to determine whether a cell is truly wild-type for a given mutation. In addition, dropout reduces the sensitivity of mutation detection by a factor of two. Partial transcript coverage is specific to end-biased platforms such as the Chromium platform, and also limits the sensitivity of variant detection. Moreover, coverage drops non-linearly across the length of the transcript, so some variants are much more easily detectable than others. The utility of this approach therefore depends on the specific mutational composition of the sample in question, and will likely perform better for other tumor types, almost all of which have higher mutation burdens than AML.

A number of other approaches to identify expressed mutations in single-cell RNA-sequencing data have been described^3–8,10–12,14,17–21^. Each method has different strengths and weaknesses that should influence the choice of platform for a specific experimental question. Key variables include library insert size, end-bias, and complexity, sequencing depth and read length, dropout rate, and throughput. Furthermore, technologies that enable simultaneous DNA and RNA sequencing of single cells, such as G&T-seq^50^, may become very powerful with increased throughput. The rapid pace of technological advancement in this area will likely increase the power of scRNA-seq to identify and distinguish among different sources of transcriptional heterogeneity in primary tumor samples.

## Methods

### Ethical approval and consent

Samples were obtained as part of a study that was approved by the Human Research Protection Office at Washington University School of Medicine (HRPO # 201011766). All the patients provided written informed consent that permitted whole-genome sequencing, in accordance with a protocol that was approved by the institutional review board at the Washington University School of Medicine.

### eWGS, germline SNP detection, and somatic variant detection

For each case, we performed enhanced whole-genome sequencing (eWGS) on bone marrow and matched normal tissue to identify germline and somatic variants. eWGS combines whole-genome sequencing with targeted exon capture to yield high coverage (~150×) of the exome, and lower genome-wide coverage in the tumor (~45×) and normal (~25×) samples. Using a previously described protocol^25^, eWGS sequencing libraries, including WGS libraries (350 bp inserts) and targeted libraries (250 bp inserts), were constructed with a KAPA HTP kit on a SciClone instrument. Targeted libraries were captured with the IDT exome reagent spiked with AML recurrently mutated genes^51^ (~40 Mb). These were sequenced on an Illumina HiSeq4000, producing ~150X coverage of each enhanced region. Sequence data were aligned to reference sequence build GRCh37-lite-build37 using BWA-MEM^52^ version 0.7.10 (params: -t 8), then merged and deduplicated using Picard version 1.113 (https://broadinstitute.github.io/picard/). Germline mutations were called using GATK HaplotypeCaller v3.5^53^ (parameters -stand_emit_conf 10 -stand_call_conf 30) and filtered using recommended parameters (–filterExpression “QD < 2.0 || FS > 60.0 || MQ < 40.0 || MQRankSum < −12.5 || ReadPosRankSum < −8.0”). SNVs were detected using an ensemble mutation calling approach^54^ that considers the union of four callers: (1) Samtools^55^ version r982 (params: mpileup -BuDs) intersected with Somatic Sniper^56^ version 1.0.4 (params: -F vcf –G -L -q 1 -Q 15) and processed through false-positive filter v1 (params:–bam-readcount- version 0.4–bamreadcount-min-base-quality 15–min-mappingquality 40–min-somatic-score 40), (2) VarScan^57^ version 2.3.6 filtered by varscan-highconfidence filter version v1 and processed through falsepositive filter v1 (params:–bamreadcount-version 0.4–bam-readcount-min-base-quality 15), (3) Strelka^58^ version 1.0.11 (params: isSkipDepthFilters = 0), and (4) Mutect^59^ v1.1.4. Indels were detected using the union of 3 callers: (1) GATK^53^ somatic-indel version 5336, (2) VarScan^57^ version 2.3.6 filtered by varscan-high-confidence- indel version v1, and (3) Strelka5 version 1.0.11 (params: isSkipDepthFilters = 0). SNVs and Indels were further filtered by removing artifacts found in a panel of 905 normal exomes^60^, removing sites that exceeded 0.1% frequency in the 1000 genomes or NHLBI exome sequencing projects, and then using a bayesian classifier (https://github.com/genome/genome/blob/master/lib/perl/Genome/Model/Tools/Validation/ IdentifyOutliers.pm) and retaining variants classified as somatic with a binomial log-likelihood of at least 10. Copy number aberrations were detected using copyCat version 1.6.10 (https://github.com/chrisamiller/copyCat) (default parameters). Somatic structural variants were detected using Manta v0.29^61^. Finally, GRCh37 genomic coordinates were translated into GRCh38 coordinates using the “liftover” utility provided by the UCSC Genome Browser (http://genome.ucsc.edu/)^62^. Sublconal architecture was inferred using the SciClone algorithm^26^.

### Bulk RNA-sequencing

RNA libraries were prepared using the TruSeq stranded kit, sequenced on the Illumina HiSeq platform, and aligned as described previously^54^. Expression quantification was performed using Kallisto 0.43.1^63^ and transcripts from ensembl version 74.

### Flow sorting for live cells for scRNA-seq

Cryovials of AML cells were thawed as follows: while 9 ml of Fetal Bovine Serum (FBS) was allowed to come to ~24 °C, AML cryovials were removed from liquid nitrogen, and warmed in a 37 °C water bath until the cells began to thaw. After 1 min, 1 ml of room temperature FBS was added to the warming cryovial with a P1000 pipet tip and allowed to mix with thawing cells. The freshly added FBS was removed from the cell pellet and transferred back to the FBS stock. This process was repeated 3–4 times until all cells from the cryovial could be poured directly into the FBS stock. The empty cryovial was rinsed once more with the FBS mixture. Cells were then pelleted by centrifugation at 300G for 5 min and resuspended in Phosphate-buffered saline (PBS) at a concentration of 1 × 10^6^ cell/ml in 1x PBS. Cells were then pipetted through a 70-µm filter into a 5-ml tube for sorting. Cells were then stained with 1 µl 7-AAD per 1 ml of cells for 30 min at 4 °C. If cell viability was ≤85%, stained cells were filtered through a 40-µM Flowmi cell strainer (Miltenyi), flow sorted, and gated using the FACS Chorus software (BD Biosciences).

### 5-prime single-cell RNA library construction and sequencing

Cells were processed using the 10x Genomics Chromium Controller and the Chromium Single Cell 5′ Library & Gel Bead Kit (PN 1000006) following the standard manufacturer’s protocols (https://tinyurl.com/y96l7lns). Two technical replicates were run in parallel for each sample. In brief, between 14,000 and 21,000 live cells were loaded onto the Chromium controller in an effort to recover between 10,000 and 15,000 cells for library preparation and sequencing. Gel beads were prepared according to standard manufacturer’s protocols. Oil partitions of single-cell + oligo coated gel beads (GEMs) were captured and reverse transcription was performed, resulting in cDNA tagged with a cell barcode and unique molecular index (UMI). Next, GEMs were broken and cDNA was amplified and quantified using an Agilent Bioanalyzer High Sensitivity chip (Agilent Technologies).

To prepare the final libraries, amplified cDNA was enzymatically fragmented, end-repaired, and polyA tagged. Fragments were then size selected using SPRIselect magnetic beads (Beckman Coulter). Next, Illumina sequencing adapters were ligated to the size-selected fragments and cleaned up using SPRIselect magnetic beads (Beckman Coulter). Finally, sample indices were selected and amplified, followed by a double sided size selection using SPRIselect magnetic beads (Beckman Coulter). Final library quality was assessed using an Agilent Bioanalyzer High Sensitivity chip. Samples were then sequenced on the Illumina NovaSeq with a target of 150,000 reads/cell (2 × 150 paired end reads), yielding a median per-library depth of 192,427 reads per cell.

### Evaluating transcript coverage as a function of distance

Transcript alignment, counting, and inter-library normalization were performed using the Cell Ranger pipeline (10x Genomics, default settings, Version 2.1.1, GRCh38 reference)^64^. For the genes *TP53*, *NPM1*, *GATA2*, and *DNMT3A*, the depth at each transcript was evaluated using both scRNA-seq data as well as bulk RNA-seq data. For each gene, a canonical isoform was chosen by consulting the APPRIS database^65^ (ENST00000445888.6, ENST00000296930.9, ENST00000341105.6, and ENST00000264709.7 respectively). For the scRNA-seq data, the number of unique barcode/UMI pairs was counted at each position. For the bulk RNA-seq data, the tool bamCoverage^66^ was used to generate a wiggle file over the transcript at 1 bp bin size. The resulting tracks were visualized using the UCSC Genome Browser^67^. To reduce visual noise from intergenic reads, positions not overlapping the canonical isoform were not considered. Coverage plots for all mutated genes in this study are provided at https://github.com/genome/scrna_mutations.

To evaluate transcriptome-wide coverage, we used the annotation set GENCODE V27 to extract 20,090 genes with only one annotated isoform between 250 bp and 11,000 bp, with an average size of 1569 bp and median size of 829 bp. Restricting to single isoform genes reduced noise related to alternative transcription start (TSS) and stop (TTS) sites. For each transcript in each sample in this study, single-cell transcriptome-wide coverage was quantified by counting the number of unique barcode/UMI pairs seen across the whole transcript. Then, for each position along the transcript, the number of unique pairs was divided by this total. This value was calculated as distance from the TSS for 5′ kit data, and distance from the TTS for 3′ kit data. To plot the results, the average value across all transcripts for all samples was calculated at each position. For shorter transcripts, positions with no data were not included in the average. The plot was also truncated to 10,000 bp to avoid edge effects related to the transcript selection process. Coverage plots were generated using the Gviz^68^ and BiomaRt^69^ R packages, versions 1.22.3 and 2.34.2 respectively. For each locus, both coding and non-coding exonic nucleotides were considered at a 1 bp bin size. Gene region tracks were retrieved directly from Ensembl v93. scRNA total read coverage was generated using bamCoverage, part of the deepTools package^66^, and scRNA cell barcode coverage can be found at https://github.com/genome/scrna_mutations.

### Copy number analysis

Gene expression matrices were analyzed with the CONICSmat package for R^19^. The default filtering and normalization procedures were followed, as outlined in https://goo.gl/tFYLEh. The mixture model results were obtained, then restricted to regions of known copy number events from the eWGS with the best log-likelihood scores from the modeling: For sample 809653, these were chromosomes 1p and 7q. The z-scored posterior probabilities were clustered, using *k* = 4, and cell barcodes from the three clusters containing one or more of the expected events were gathered and visualized on the expression t-SNE projection.

### Single-cell mutation identification and analysis

We processed the aligned sequence data using a Pysam^70^-based tool (https://github.com/sridnona/cb_sniffer). For each cell barcode in the filtered Cell Ranger barcode list, and each somatic variant in the eWGS data, variant bases were identified, excluding exclude those with base quality and mapping quality <1. Only reads that had both a Chromium Cellular Barcode (CB) tag and a Chromium Molecular Barcode (UB) tag were included. We then obtained the cell-associated tag for downstream analysis of UMIs. In rare cases where duplicate reads existed for a given UB and the base at the mutant position was not identical across all reads, we selected the most common base if it was present in at least 75% of the reads; otherwise all reads in the group corresponding to that UB were discarded. We rarely observed such discordant reads (for example, they occurred in 782328 at a frequency of 4/6218, or 0.06%).

Several variants required additional steps in order to accurately identify mutant cells: Manual review revealed that two small indels in repetitive regions (*CEBPA* (19:33301989–33301990) and *NPM1* (5:171410538–171410546)) were frequently misaligned to several adjacent bases. This was resolved by parsing the bam cigar string to identify reads containing insertions or deletions at the appropriate locations using an additional Pysam-based tool (https://github.com/genome/scrna_mutations/tree/master/misc_scripts), which extracts overlapping reads using SAMtools ‘view’^55^. In addition, the large size of the characteristic large internal tandem duplication (ITD) in *FLT3* resulted in incorrect alignment of many variant-containing reads. To address this, we created a contig containing the variant sequence (±250 bp), appended it to the transcriptome reference, and realigned the scRNA data to the expanded reference. Barcodes from reads uniquely aligning to the mutant *FLT3* sequence were then extracted. Similarly, the *NUP98*-*NSD1* fusion in 508084 was detected by appending the fusion transcript to the input GTF file, then using kallisto^63^ and its companion tool, pizzly, to identify fusion-supporting transcripts.

After using SciClone^26^ to assign each somatic variant to a subclone, we assigned mutation-containing cells (“mutant cells”) to their corresponding subclones. Cell-variant assignment can now also be performed in an automated manner using the VarTrix tool (https://github.com/10xgenomics/vartrix).

### Read-based and cell-based metrics

The read-based metric “single-cell Variant Allele Frequency,” or scVAF, was defined for each variant discovered in the eWGS data as the number of mutant reads divided by the total number of reads mapping to the variant position in the scRNA-seq data. The two cell-based metrics were (1) Mutant Cell Fraction (MCF) and (2) Mutant Cell Detection Rate (MCDR)). The MCF for each variant was defined as *M*/*T*, where *T* is the number cells having coverage at the mutant site, and *M* is the number of cells having at least one mutant read at that site. The Mutant Cell Detection Rate (MCDR) was defined as the ratio of observed mutant cells to the number of expected mutant cells; for variants with coverage in bulk RNA-seq data, the number of expected mutant cells is twice the eWGS VAF.

### Mutation detection in normal bone marrow samples

We used cb_sniffer to search four normal bone marrow samples for the somatic mutations discovered by eWGS. These samples had been previously generated using the methods described above and the Chromium Single Cell 3′ Library & Gel Bead Kit (v2).

### scRNA-seq expression analysis and mutation integration

Transcript alignment, counting, and inter-library normalization were performed using the Cell Ranger pipeline (10x Genomics, default settings, Version 2.1.1). Using the Seurat R package^33^, cells that contained fewer than 10 expressed genes, more than 50% ribosomal transcripts, or more than 10% mitochondrial transcripts were removed. Genes that were expressed in fewer than three cells were also removed. For each cell, expression of each gene was normalized to the sequencing depth of the cell, scaled to a constant depth (10,000), and log-transformed. Variable genes were selected (x.low.cutoff = 0.0125, x.high.cutoff = 5, y.cutoff = 0.5, default settings otherwise). Principal component analysis was performed on the variable genes, and the optimal number of principal components (PCs) for each sample was chosen using a combination of elbow plots, jackstraw resampling, and PC expression heatmaps (508084: 6, 548327: 8, 721214: 5, 782328: 7, 809653: 6, 809653 AML cells: 6). PCs were used for dimensionality reduction if they explained at least 2% of the variance; were statistically significant according to jackstraw resampling; exhibited consistent expression variation in heatmaps; and were not composed entirely of ribosomal, mitochondrial, or immune genes. Dimensionality reduction and visualization were performed with the t-SNE algorithm (Seurat implementation) using the PCs selected above. Unsupervised graph-based clustering of cells was performed using the indicated PCs, with resolution = 0.7. Cell cycle phase was determined using methodology provided in Seurat, based on relative expression of phase-specific genes^6^. The distribution of mutations on the t-SNE projection was robust to filtering for mitochondrial and ribosomal transcripts, the number of PCs used, the clustering resolution, and normalization for cell cycle phase. The mutation distribution was also robust to the particular implementation of the t-SNE algorithm, with the Seurat and Cell Ranger implementations giving consistent results. To assess the relationship between mutation distribution and expression of the mutated gene, we colored each cluster in each t-SNE plot according to the expression-normalized mutant cell fraction (mutant cell fraction divided by the average expression of the mutant gene in that cluster).

Mutation-expressing cells were analyzed in isolation using analogous methods, with the exception that fewer PCs were required to capture the variability in the data (508084: 4, 548327: 3, 721214: 6, 782328: 7, 809653: 6).

### Expression heatmaps

An expression heatmap was generated for each sample by selecting the top 10 genes in each of the top 20 PCs. To connect heterogeneity to the graph-based clusters, and to examine relationships among clusters, we averaged the expression of each gene within each cluster, and hierarchically clustered the results. For the analogous analysis performed on mutant cells in isolation, we used the top 20 genes from each of the top *n* PCs, where *n* was chosen separately for each sample to minimize noise (508084: 4, 548327: 3, 721214: 6, 782328: 7, 809653: 6).

### Lineage inference and AML cell identification

Cell-type inference was performed in an unsupervised, marker-free manner by training a nearest-neighbor algorithm on expression data from the DMAP database^27^, using Spearman correlation as the distance metric. Using this approach, cells that co-cluster by graph-based clustering tend to have the same inferred lineage and express the corresponding cell-type markers (when known). In the case of AML cells, the assigned lineage represents the normal lineage to which the AML cell is most transcriptionally similar. To identify AML cells in highly heterogeneous samples (549327 and 809653), a one-sided Fisher exact test was used to identify cell clusters that were enriched for somatic mutations (*p* ≤ 0.05). In cases where most cells are AML cells, normal cell clusters were identified using a one-sided Fisher exact test for under-enrichment (*p* ≤ 0.05).

### *GATA2*^R361C^-associated expression signatures

Each cell containing a *GATA2*^R361C^ mutation was assigned to an expression cluster. In order to incorporate expression information from all cells in the data set, including those of undetermined genotype, and to make use of quantitative information about local mutation density, we used a regression model to identify genes whose expression depends on mutant cell concentration. For each gene *i*, multiple regression was used to quantify the relationship between mean expression (*E*_*i*_) and *GATA2*^R361C^ mutant cell fraction (*m*) across the 12 AML clusters, while controlling for mean cluster-wise *GATA2* expression (*g*):

2 $$E_i = x_i + y_im + z_ig$$

We selected genes whose *q-*value (F-test with Benjamini-Hochberg correction for multiple hypotheses) for *y*_*i*_ was at most 0.05. To consider the entire subclone containing the *GATA2* mutation, we performed this procedure using cells containing any detected mutation in that subclone (*GATA2*^R361C^ or *TIMM17B*^L122fs^), without the correction for *GATA2* expression. To analyze the mutant-only data, we used a Wilcoxon test to perform a binary comparison of mutation-rich clusters to mutation-poor clusters, with analogous *p*-value correction and cutoffs.

### Identification and analysis of the *VIM* regulon

Genes that exhibited subclone-specific expression (above) were compared to genes whose expression was highly *VIM*-correlated in TCGA (*q* < 0.001, Pearson correlation, Benjamini-Hochberg correction for multiple hypotheses). Genes in the intersection were considered part of the “*VIM* regulon,” and Toppfun^29^ was used to characterize their functional enrichment.

### Orthogonal confirmation of *GATA2*^R361C^ signature

Primary, human AML peripheral blood and bone marrow aspirate samples (721214) were thawed from cryopreserved stocks and labeled with CD99 FITC-conjugated antibody (clone 3B2/TA8, ThermoFisher) in staining buffer (2% fetal bovine serum, 0.25 mM EDTA in PBS) for 30 min at 4 °C followed by viability dye for 5 min at room temperature (SytoxBlue, ThermoFisher). Live cells were analyzed using a Sony SY3200 Synergy flow cytometer, gated for sorting on the top 15% and bottom 15% with respect to *CD99* expression, and collected for analysis. Genomic DNA was prepared with the QIAmp DNA micro kit (Qiagen) according to the manufacturer’s protocol. Targeted sequencing was achieved by generating amplicons to capture mutations at *DNMT3A*^R882^ (forward: CGCAAAATACTCCTTCAGCG, reverse: TTTCTCCCCCAGGGTATTTG) and *GATA2*^R361^ (forward: TGTGCAGCTTGTAGTAGAGG, reverse: TGAGATTTAGCCCTCCTTGAC). Amplicons were indexed and spiked into 2x150 dual indexed runs on an Illumina MiniSeq sequencer. FastQC^71^ was used for quality analysis of sequenced reads (FASTQ files). Reads were checked for contamination, adapter sequences and base quality, then aligned against human reference sequence (GRCh37) using bwa (version 0.7.15)^72^. Varscan2^57^ was used to identify SNVs and calculate variant allele frequencies (VAF).

### *RNF10*^NULL^ and *CEBPA*^R142fs^ mutant expression signatures

We used a Wilcoxon rank sum test to perform a binary comparison of mutation-rich clusters to mutation-poor AML cell clusters, with *p*-value correction and cutoffs as described above. Mutation-rich clusters were significantly enriched for mutations (*p* = 0.00085 (*CEBPA*^R142fs^) and *p* = 0.0044 (*RNF10*^NULL^), Fisher Exact test).

### Functional enrichment

Functional enrichment analyses were performed using ToppFun (https://toppgene.cchmc.org/enrichment.jsp)^29^.

### Reporting summary

Further information on research design is available in the Nature Research Reporting Summary linked to this article.

## Supplementary information

Supplementary Information

Description of Additional Supplementary Files

Supplementary Data 1

Supplementary Data 2

Supplementary Data 3

Supplementary Data 4

Supplementary Data 5

Reporting Summary

## Data Availability

Enhanced whole-genome sequence (eWGS), bulk RNA-sequence, and single-cell RNA-sequence (scRNA-seq) data generated during the current study are available in dbGaP (https://www.ncbi.nlm.nih.gov/gap/) with the primary accession code phs000159. The SRA IDs for this study are: SRR7904017, SRR7904018, SRR7904019, SRR7904020, SRR7910353, SRR7910351, SRR7910349, SRR7904016, SRR7903979, SRR7825447, SRR7825459, SRR7825446, SRR7825444, SRR7825491, SRR7825473, SRR7825453, SRR7825466, SRR7825499, SRR7825482, and SRR7939318. Processed single-cell RNA-seq and mutation data pertaining to AML samples and normal bone marrow are also available [10.5281/zenodo.3345981]. All the other data supporting the findings of this study are available within the article and its supplementary information files and from the corresponding author upon reasonable request. A reporting summary for this article is available as a Supplementary Information file.

## Code availability

Single-cell mutation identification was performed using Pysam-based tools available at https://github.com/sridnona/cb_sniffer and https://github.com/genome/scrna_mutations/tree/master/misc_scripts. A comparable tool provided by 10x Genomics is available at https://github.com/10xgenomics/vartrix.
