# Author Correction: A general approach for detecting expressed mutations in AML cells using single cell RNA-sequencing

**DOI:** 10.1038/s41467-022-31995-w

**Published:** 2022-07-21

**Authors:** Allegra A. Petti, Stephen R. Williams, Christopher A. Miller, Ian T. Fiddes, Sridhar N. Srivatsan, David Y. Chen, Catrina C. Fronick, Robert S. Fulton, Deanna M. Church, Timothy J. Ley

**Affiliations:** 1 Division of Oncology, Washington University School of Medicine, St. Louis, MO USA; 2 McDonnell Genome Institute, Washington University School of Medicine, St. Louis, MO USA; 3 10x Genomics, Inc., Pleasanton, CA USA; 4 Division of Dermatology, Washington University School of Medicine, St. Louis, MO USA; 5 Inscripta, Inc., Boulder, CO USA; 6 Department of Genetics, Washington University School of Medicine, St. Louis, MO USA

**Keywords:** Cancer genomics, Acute myeloid leukaemia, Tumour heterogeneity, Data integration

Correction to: *Nature Communications* 10.1038/s41467-019-11591-1, published online 14 August 2019.

The original version of this article contained errors in Tables 1 and 2, which don’t affect the conclusions. In Table 1, several numbers were incorrectly given in columns corresponding to the samples #548327, #721214 and #809653. The previous version of Table 1 was:

Original Table 1. Overview of mutation discovery and detection in eWGS and scRNA-seq data.

**Table:** 

Sample	508084	548327	721214	782328	809653	Mean [SD]
No. cells	14,964	11,620	20,474	21,731	21,038	17,965 [3979]
Reads/cell	192,427	346,965	176,035	214,284	189,751	223,892 [62,745]
Reads mapped confidently to genome (%)	84.9	87.2	92.2	79.8	90	87 [4.29]
Reads mapped confidently to transcriptome (%)	64.2	63.7	73	61.9	68.8	66 [4.04]
Median genes detected per cell	2405	1383	2260	1376	1829	1851 [428]
Total genes detected	22,645	22,503	23,376	25,389	23,102	23,403 [1041]
WGS variants	19	13	41	31	28	26.4
Expressed WGS variants (bulk)	10	5	18	7	8	9.6
scRNA-seq variants	8	7	17	12	8	10.4
% expressed WGS variants discovered in scRNA-seq	80%	140%	94%	170%	100%	117%
Total Mutant cells	669	6042	8200	3354	386	3732
Mutant cells per variant	13–453	1–3012	1–3944	1–2619	1–207	3.4–2047
Mutant cells per variant (median)	32	48	30	111	21.5	48.5
Key WGS variants (no. cells with scRNA-seq coverage at variant position)	*IKBKB*^*V616M*^ (150) *FLT3-ITD* (707) *NUP98-NDS1* *(1)*	*IDH1*^*R132H*^ (118) *NPM1*^*W288fs*^ (5591) *SRSF2*^*P95H*^ (2349)	*DNMT3A* ^*R882H*^ (409) *FLT3-ITD* (479) *FLT3*^*F612L*^ (306) *NPM1*^*W288fs*^ (11,672) *GATA2*^*R361C*^ (1629)	*NRAS*^*G12S*^ (949) *NRAS*^*G12D*^ (951) *U2AF1*^*S34F*^ (4509)	*NRAS*^*G12D*^ (1412) *TP53*^*E286G*^ (239) *CEBPA*^*R142fs*^ (84)	N/A
Additional variants with expression signature (number of cells with coverage)	*RNF10 (103)*			*NAGLU*^*E634K*^ *(216)*		N/A

The correct version appears as:

Revised Table 1. Overview of mutation discovery and detection in eWGS and scRNA-seq data.

**Table:** 

Sample	508084	548327	721214	782328	809653	Mean [SD]
No. cells	14,964	11,620	20,474	21,731	21,038	17,965 [3979]
Reads/cell	192,427	346,965	176,035	214,284	189,751	223,892 [62,745]
Reads mapped confidently to genome (%)	84.9	87.2	92.2	79.8	90	87 [4.29]
Reads mapped confidently to transcriptome (%)	64.2	63.7	73	61.9	68.8	66 [4.04]
Median genes detected per cell	2405	1383	2260	1376	1829	1851 [428]
Total genes detected	22,645	22,503	23,376	25,389	23,102	23,403 [1041]
WGS variants	19	13	41	31	28	26.4
Expressed WGS variants (bulk)	10	5	18	7	8	9.6
scRNA-seq variants	8	7	17	12	8	10.4
% expressed WGS variants discovered in scRNA-seq	80%	140%	94%	170%	100%	117%
Total mutant cells	669	3571	4975	3354	361	2586
Mutant cells per variant	13–453	1–3012	1–3944	1–2619	1–207	3.4–2047
Mutant cells per variant (median)	32	48	30	111	21.5	48.5
Key WGS variants (no. cells with scRNA-seq coverage at variant position)	*IKBKB*^*V616M*^ (150) *FLT3-ITD* (707) *NUP98-NDS1* *(1)*	*IDH1*^*R132H*^ (118) *NPM1*^*W288fs*^ (5591) *SRSF2*^*P95H*^ (2349)	*DNMT3A* ^*R882H*^ (409) *FLT3-ITD* (479) *FLT3*^*F612L*^ (306) *NPM1*^*W288fs*^ (11,672) *GATA2*^*R361C*^ (1629)	*NRAS*^*G12S*^ (949) *NRAS*^*G12D*^ (951) *U2AF1*^*S34F*^ (4509)	*NRAS*^*G12D*^ (1412) *TP53*^*E286G*^ (239) *CEBPA*^*R142fs*^ (84)	N/A
Additional variants with expression signature (number of cells with coverage)	*RNF10 (103)*			*NAGLU*^*E634K*^ *(216)*		N/A

In Table 2, several numbers were incorrectly given in columns corresponding to the samples #548327, #721214, and #809653. The previous version of Table 2 was:

Original Table 2. Frequency of cells containing multiple mutations in each case.

**Table:** 

Sample	508084	548327	721214	782328	809653
Total mutant cells	669	6042	8200	3354	396
1 mutation	658 (98%)	5290 (88%)	7702 (94%)	3176 (95%)	386 (97%)
2 mutations	11 (1.6%)	734 (12%)	477 (5.8%)	171 (5.1%)	10 (2.5%)
3 mutations	0	18 (0.29%)	20 (0.24%)	7 (0.21%)	0
4 mutations	0	0	1 (0.012%)	0	0

The correct version appears as:

Revised Table 2. Frequency of cells containing multiple mutations in each case.

**Table:** 

Sample	508084	548327	721214	782328	809653
Total mutant cells	669	3571	4975	3354	361
1 mutation	658 (98%)	3280 (92%)	4694 (94%)	3176 (95%)	354 (98%)
2 mutations	11 (1.6%)	460 (13%)	268 (5.4%)	171 (5.1%)	7 (1.9%)
3 mutations	0	11 (0.31%)	12 (0.24%)	7 (0.21%)	0
4 mutations	0	0	1 (0.02%)	0	0

These errors have been corrected in the PDF and HTML versions of the article.

