# Supplementary material for: A general approach for detecting expressed mutations in AML cells using single cell RNA-sequencing
Source: Nat Commun. 2019 Aug 14;10:3660. doi: 10.1038/s41467-019-11591-1 (PMC6694122; doi:10.1038/s41467-019-11591-1)
Supplement: Supplementary file 1 — Supplementary Information [file 41467_2019_11591_MOESM1_ESM.pdf]

Supplementary Information for:

A general approach for detecting expressed mutations in AML cells using single cell RNA-sequencing

Petti et al.

Supplementary Figures and Legends

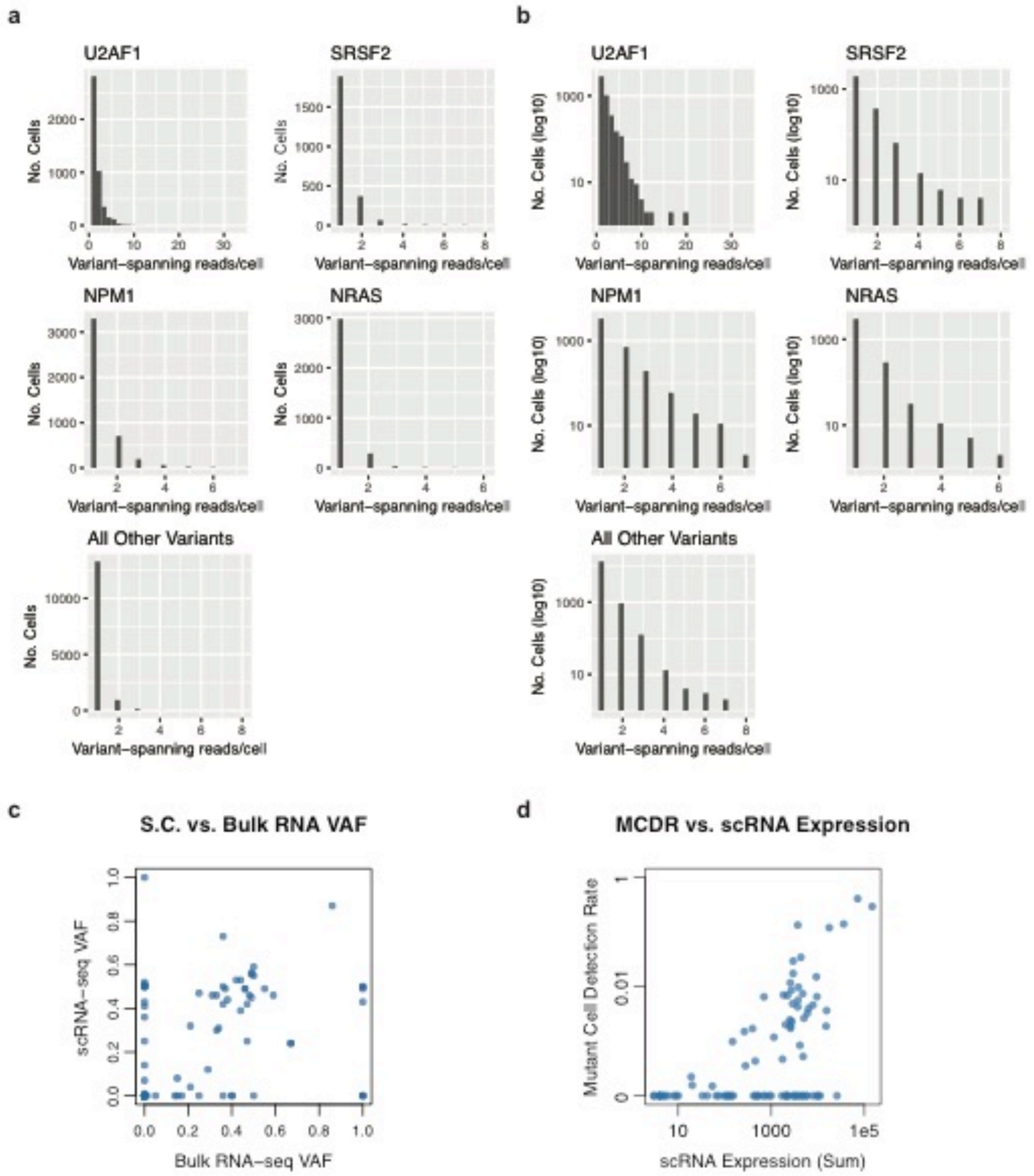

Supplementary Figure 1. Additional performance metrics for single-cell variant detection. (a) Distribution of variant-spanning reads for mutations in the indicated gene(s). (b) Log-scale distribution of variant-spanning reads for mutations in the indicated gene(s). (c) Relationship

between single-cell and bulk RNA-seq VAF. (d) Mutant Cell Detection Rate as a function of gene expression level in the single-cell data.

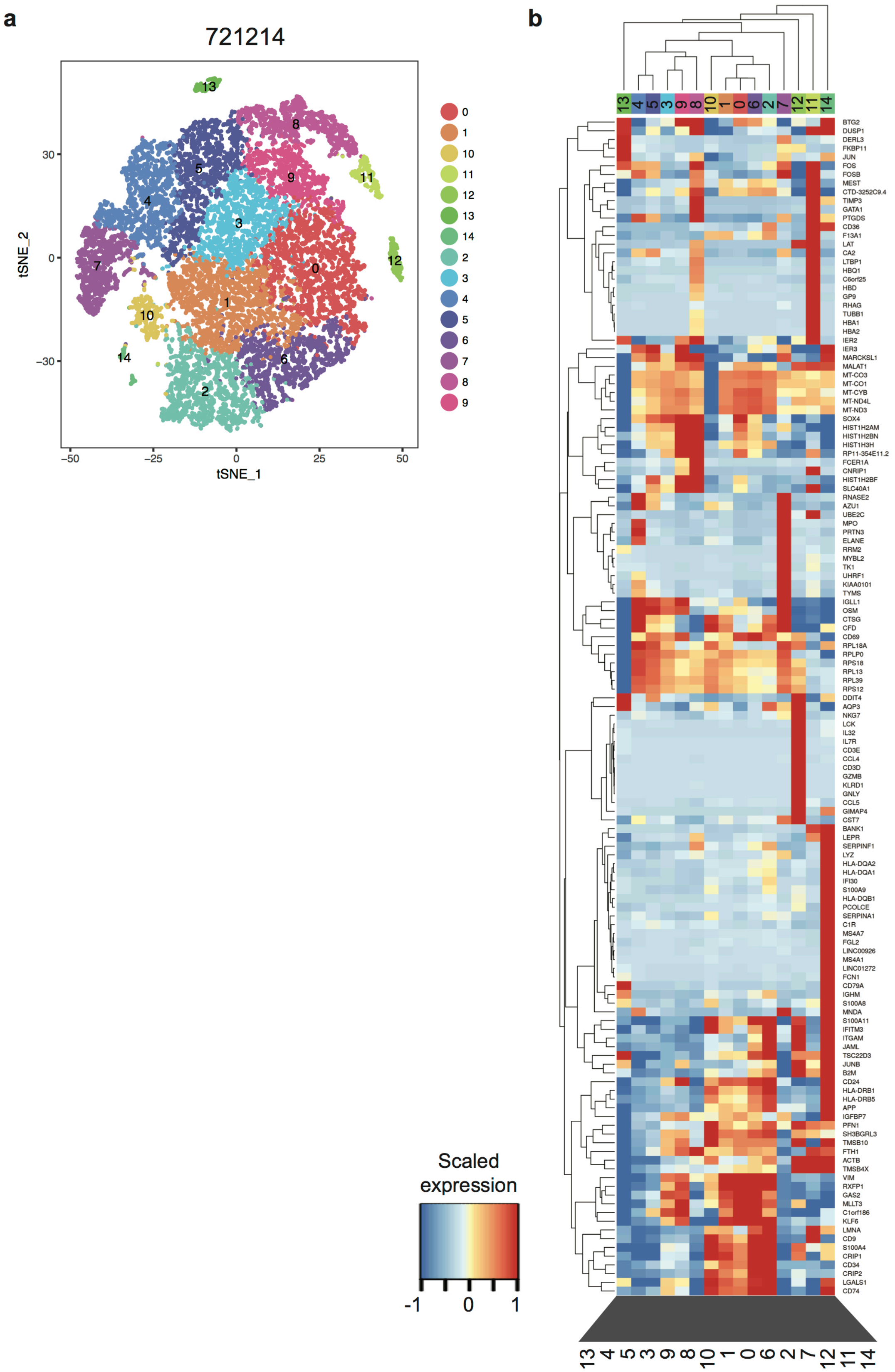
a
721214
tSNE_2
tSNE_1
30
0
−30
−50
−25
0
25
50
0
1
10
11
12
13
14
2
3
4
5
6
7
8
9
b
13 4 5 3 9 8 10 1 0 6 2 7 12 11 14
BTG2
DUSP1
DERL3
FKBP11
JUN
FOS
FOSB
MEST
CTD-3252C9.4
TIMP3
GATA1
PTGDS
CD36
F13A1
LAT
CA2
LTBP1
HBQ1
C6orf25
HBD
GP9
RHAG
TUBB1
HBA1
HBA2
IER2
IER3
MARCKSL1
MALAT1
MT-CO3
MT-CO1
MT-CYB
MT-ND4L
MT-ND3
SOX4
HIST1H2AM
HIST1H2BN
HIST1H3H
RP11-354E11.2
FCER1A
CNRIP1
HIST1H2BF
SLC40A1
RNASE2
AZU1
UBE2C
MPO
PRTN3
ELANE
RRM2
MYBL2
TK1
UHRF1
KIAA0101
TYMS
IGLL1
OSM
CTSG
CFD
CD69
RPL18A
RPLP0
RPS18
RPL13
RPL39
RPS12
DDIT4
AQP3
NKG7
LCK
IL32
IL7R
CD3E
CCL4
CD3D
GZMB
KLRD1
GNLY
CCL5
GIMAP4
CST7
BANK1
LEPR
SERPINF1
LYZ
HLA-DQA2
HLA-DQA1
IFI30
S100A9
HLA-DQB1
PCOLCE
SERPINA1
C1R
MS4A7
FGL2
LINC00926
MS4A1
LINC01272
FCN1
CD79A
IGHM
S100A8
MNDA
S100A11
IFITM3
ITGAM
JAML
TSC22D3
JUNB
B2M
CD24
HLA-DRB1
HLA-DRB5
APP
IGFBP7
PFN1
SH3BGRL3
TMSB10
FTH1
ACTB
TMSB4X
VIM
RXFP1
GAS2
MLLT3
C1orf186
KLF6
LMNA
CD9
S100A4
CRIP1
CD34
CRIP2
LGALS1
CD74
Scaled expression
-1
0
1
13 4 5 3 9 8 10 1 0 6 2 7 12 11 14

Supplementary Figure 2. Clustering and overview of expression heterogeneity in 721214. (a) t-SNE plot of scRNA-seq data, cells colored according to graph-based cluster assignment; putative AML clusters circled. (b) Hierarchical clustering of the most heavily weighted genes in each principal component, averaged within graph-based clusters. Each column corresponds to a cluster in panel a.

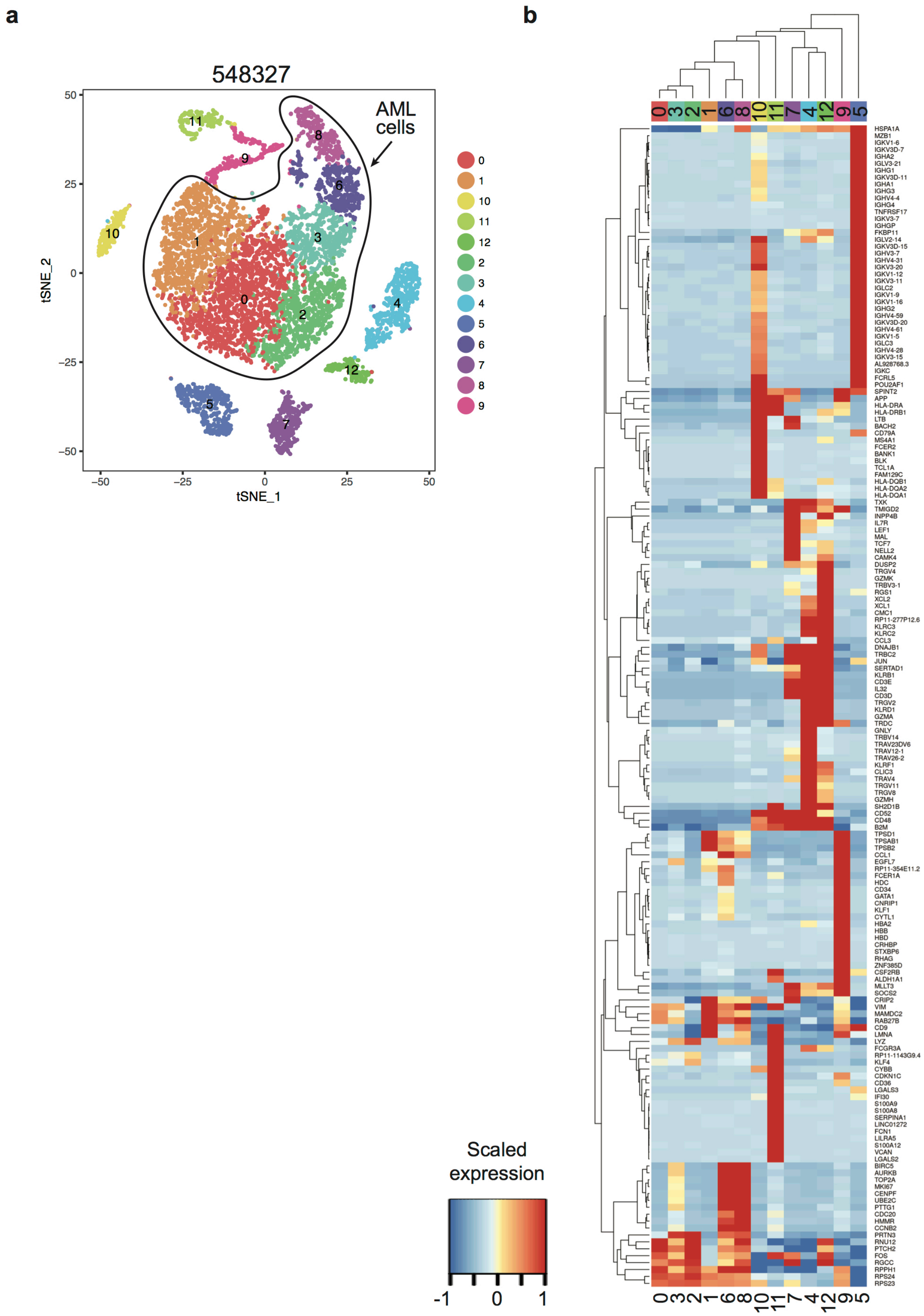
a
548327
AML cells
tSNE_2
tSNE_1
50
25
0
−25
−50
−50
−25
0
25
50
0
1
10
11
12
2
3
4
5
6
7
8
9
b
Scaled expression
-1
0
1
0 3 2 1 6 8 10 11 7 4 12 9 5
HSPA1A
MZB1
IGKV1-6
IGKV3D-7
IGHA2
IGLV3-21
IGHG1
IGKV3D-11
IGHA1
IGHG3
IGHV4-4
IGHG4
TNFRSF17
IGKV3-7
IGHGP
FKBP11
IGLV2-14
IGKV3D-15
IGHV3-7
IGHV4-31
IGKV3-20
IGKV1-12
IGKV3-11
IGLC2
IGKV1-9
IGKV1-16
IGHG2
IGHV4-59
IGKV3D-20
IGHV4-61
IGKV1-5
IGLC3
IGHV4-28
IGKV3-15
AL928768.3
IGKC
FCRL5
POU2AF1
SPINT2
APP
HLA-DRA
HLA-DRB1
LTB
BACH2
CD79A
MS4A1
FCER2
BANK1
BLK
TCL1A
FAM129C
HLA-DQB1
HLA-DQA2
HLA-DQA1
TXK
TMIGD2
INPP4B
IL7R
LEF1
MAL
TCF7
NELL2
CAMK4
DUSP2
TRGV4
GZMK
TRBV3-1
RGS1
XCL2
XCL1
CMC1
RP11-277P12.6
KLRC3
KLRC2
CCL3
DNAJB1
TRBC2
JUN
SERTAD1
KLRB1
CD3E
IL32
CD3D
TRGV2
KLRD1
GZMA
TRDC
GNLY
TRBV14
TRAV23DV6
TRAV12-1
TRAV26-2
KLRF1
CLIC3
TRAV4
TRGV11
TRGV8
GZMH
SH2D1B
CD52
CD48
B2M
TPSD1
TPSAB1
TPSB2
CCL1
EGFL7
RP11-354E11.2
FCER1A
HDC
CD34
GATA1
CNRIP1
KLF1
CYTL1
HBA2
HBB
HBD
CRHBP
STXBP6
RHAG
ZNF385D
CSF2RB
ALDH1A1
MLLT3
SOCS2
CRIP2
VIM
MAMDC2
RAB27B
CD9
LMNA
LYZ
FCGR3A
RP11-1143G9.4
KLF4
CYBB
CDKN1C
CD36
LGALS3
IFI30
S100A9
S100A8
SERPINA1
LINC01272
FCN1
LILRA5
S100A12
VCAN
LGALS2
BIRC5
AURKB
TOP2A
MKI67
CENPF
UBE2C
PTTG1
CDC20
HMMR
CCNB2
PRTN3
RNU12
PTCH2
FOS
RGCC
RPPH1
RPS24
RPS23

Supplementary Figure 3. Clustering and overview of expression heterogeneity in 548327. (a) t-SNE plot of scRNA-seq data, cells colored according to graph-based cluster assignment; putative AML clusters circled. (b) Hierarchical clustering of the most heavily weighted genes in each principal component, averaged within graph-based clusters.

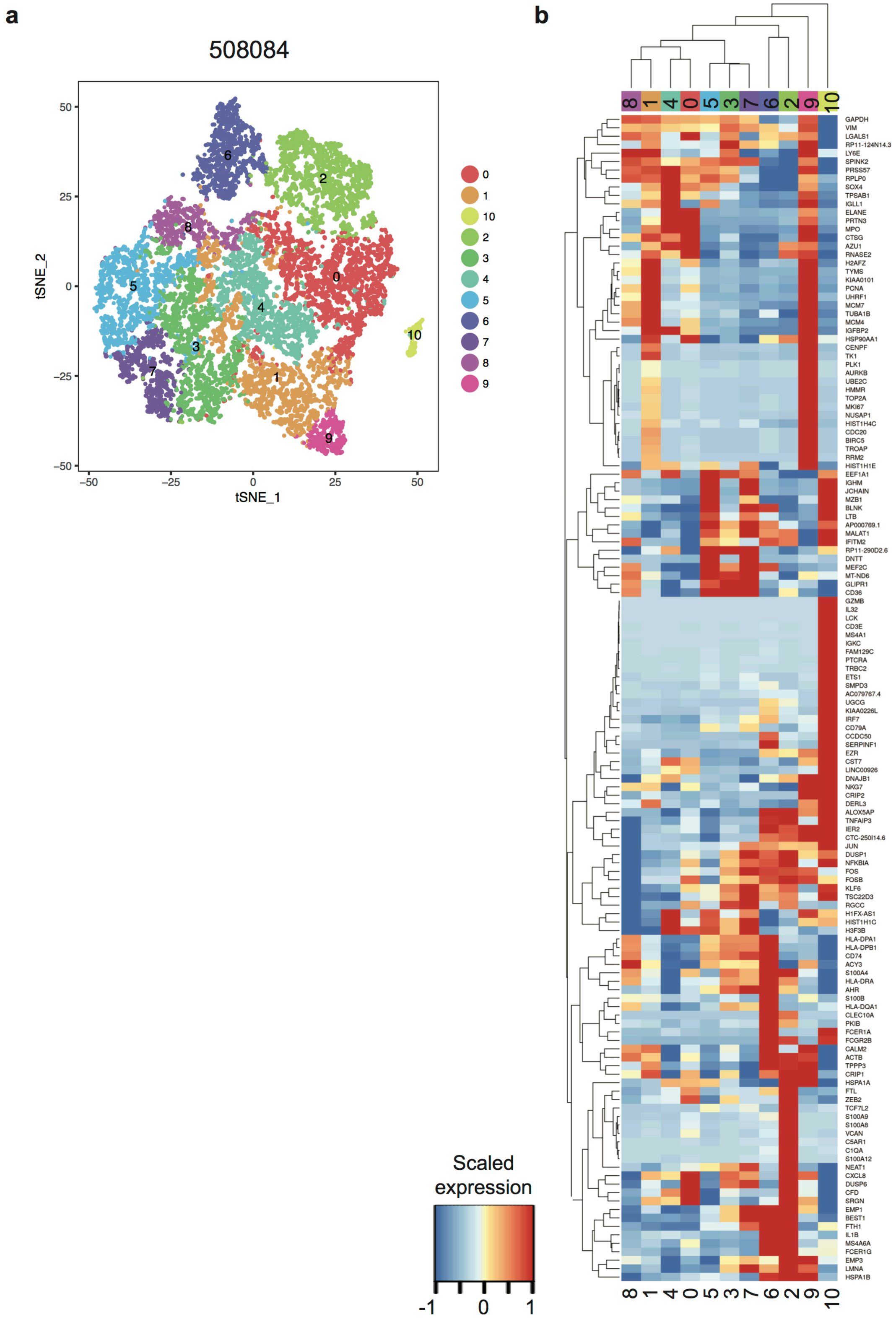
a
508084
tSNE_2
tSNE_1
50
25
0
-25
-50
0
1
10
2
3
4
5
6
7
8
9
b
8 1 4 0 5 3 7 6 2 9 10
Scaled expression
-1
0
1

Supplementary Figure 4. Clustering and overview of expression heterogeneity in 508084. (a) t-SNE plot of scRNA-seq data, cells colored according to graph-based cluster assignment; putative AML clusters circled. (b) Hierarchical clustering of the most heavily weighted genes in each principal component, averaged within graph-based clusters.

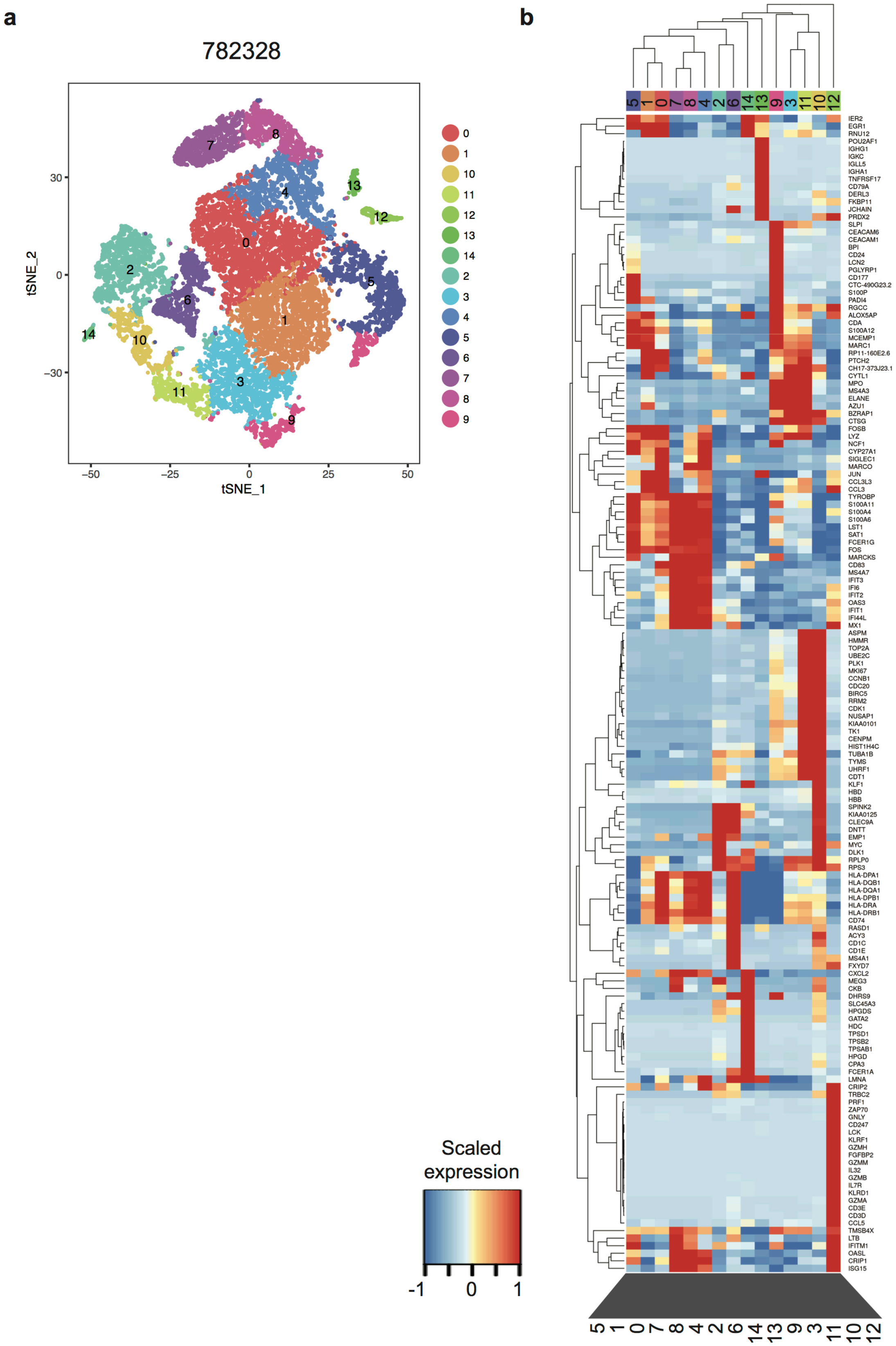
a
782328
tSNE_2
tSNE_1
30
0
–30
–50
–25
0
25
50
0
1
10
11
12
13
14
2
3
4
5
6
7
8
9
b
5 1 0 7 8 4 2 6 14 13 9 3 11 10 12
IER2
EGR1
RNU12
POU2AF1
IGHG1
IGKC
IGLL5
IGHA1
TNFRSF17
CD79A
DERL3
FKBP11
JCHAIN
PRDX2
SLPI
CEACAM6
CEACAM1
BPI
CD24
LCN2
PGLYRP1
CD177
CTC-490G23.2
S100P
PADI4
RGCC
ALOX5AP
CDA
S100A12
MCEMP1
MARC1
RP11-160E2.6
PTCH2
CH17-373J23.1
CYTL1
MPO
MS4A3
ELANE
AZU1
BZRAP1
CTSG
FOSB
LYZ
NCF1
CYP27A1
SIGLEC1
MARCO
JUN
CCL3L3
CCL3
TYROBP
S100A11
S100A4
S100A6
LST1
SAT1
FCER1G
FOS
MARCKS
CD83
MS4A7
IFIT3
IFI6
IFIT2
OAS3
IFIT1
IFI44L
MX1
ASPM
HMMR
TOP2A
UBE2C
PLK1
MKI67
CCNB1
CDC20
BIRC5
RRM2
CDK1
NUSAP1
KIAA0101
TK1
CENPM
HIST1H4C
TUBA1B
TYMS
UHRF1
CDT1
KLF1
HBD
HBB
SPINK2
KIAA0125
CLEC9A
DNTT
EMP1
MYC
DLK1
RPLP0
RPS3
HLA-DPA1
HLA-DQB1
HLA-DQA1
HLA-DPB1
HLA-DRA
HLA-DRB1
CD74
RASD1
ACY3
CD1C
CD1E
MS4A1
FXYD7
CXCL2
MEG3
CKB
DHRS9
SLC45A3
HPGDS
GATA2
HDC
TPSD1
TPSB2
TPSAB1
HPGD
CPA3
FCER1A
LMNA
CRIP2
TRBC2
PRF1
ZAP70
GNLY
CD247
LCK
KLRF1
GZMH
FGFBP2
GZMM
IL32
GZMB
IL7R
KLRD1
GZMA
CD3E
CD3D
CCL5
TMSB4X
LTB
IFITM1
OASL
CRIP1
ISG15
Scaled expression
-1
0
1
5 1 0 7 8 4 2 6 14 13 9 3 11 10 12

Supplementary Figure 5. Clustering and overview of expression heterogeneity in 782328. (a) t-SNE plot of scRNA-seq data, cells colored according to graph-based cluster assignment; putative AML clusters circled. (b) Hierarchical clustering of the most heavily weighted genes in each principal component, averaged within graph-based clusters.

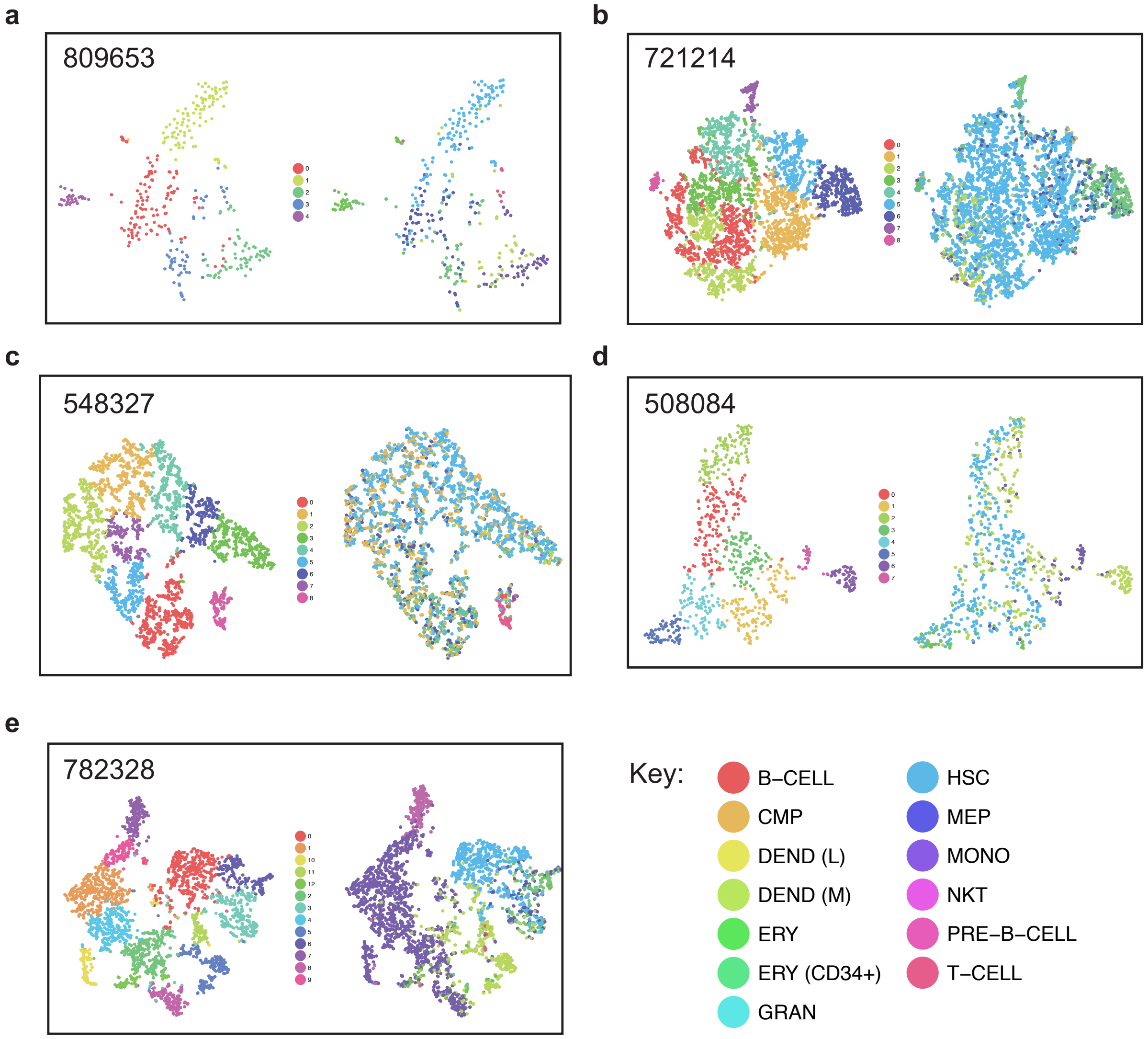

Supplementary Figure 6. Dimensionality reduction, clustering, and lineage inference of mutant cells. (a) t-SNE projection of 809653 with cells colored according to graph-based cluster assignment (left) and inferred cell lineage (right); CMP = common myeloid progenitor, DEND (L) = lymphoid dendritic cell, DEND (M) = myeloid dendritic cell, ERY = erythrocyte, ERY (CD34+) = CD34+ erythrocyte, GRAN = granulocyte, HSC = hematopoietic stem cell, MEP = myeloid-erythroid progenitor, MONO = monocytic lineage, NKT = NK-T cell. (b) 721214. (c) 548327. (d) 508084. (e) 782328.

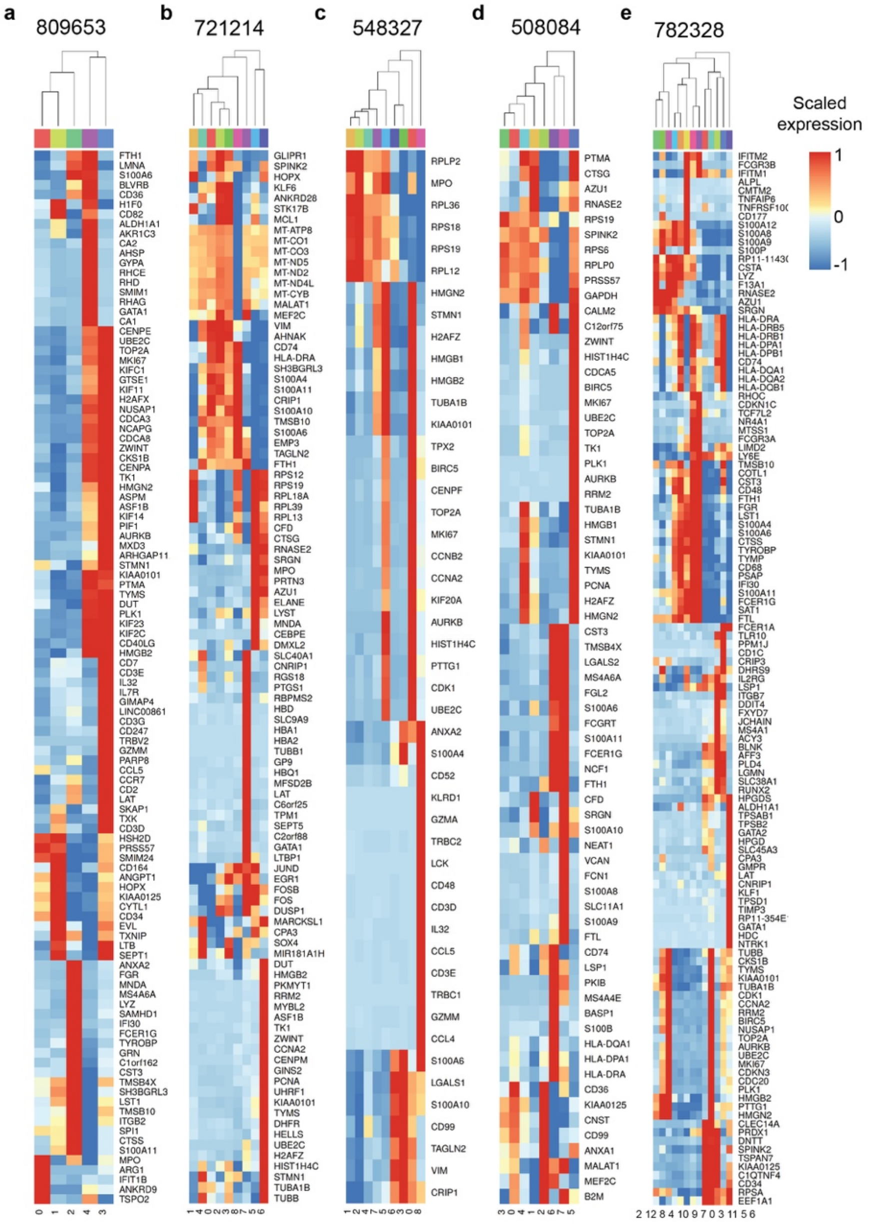

Supplementary Figure 7. Cluster-averaged gene expression profiles of highly variable genes in mutant cells. Each column corresponds to a graph-based cluster in Supp. Fig. 6. Genes were chosen based on their contributions to the top principal components (Methods). (a) 809653. (b) 721214. (c) 548327. (d) 508084. (e) 782328.

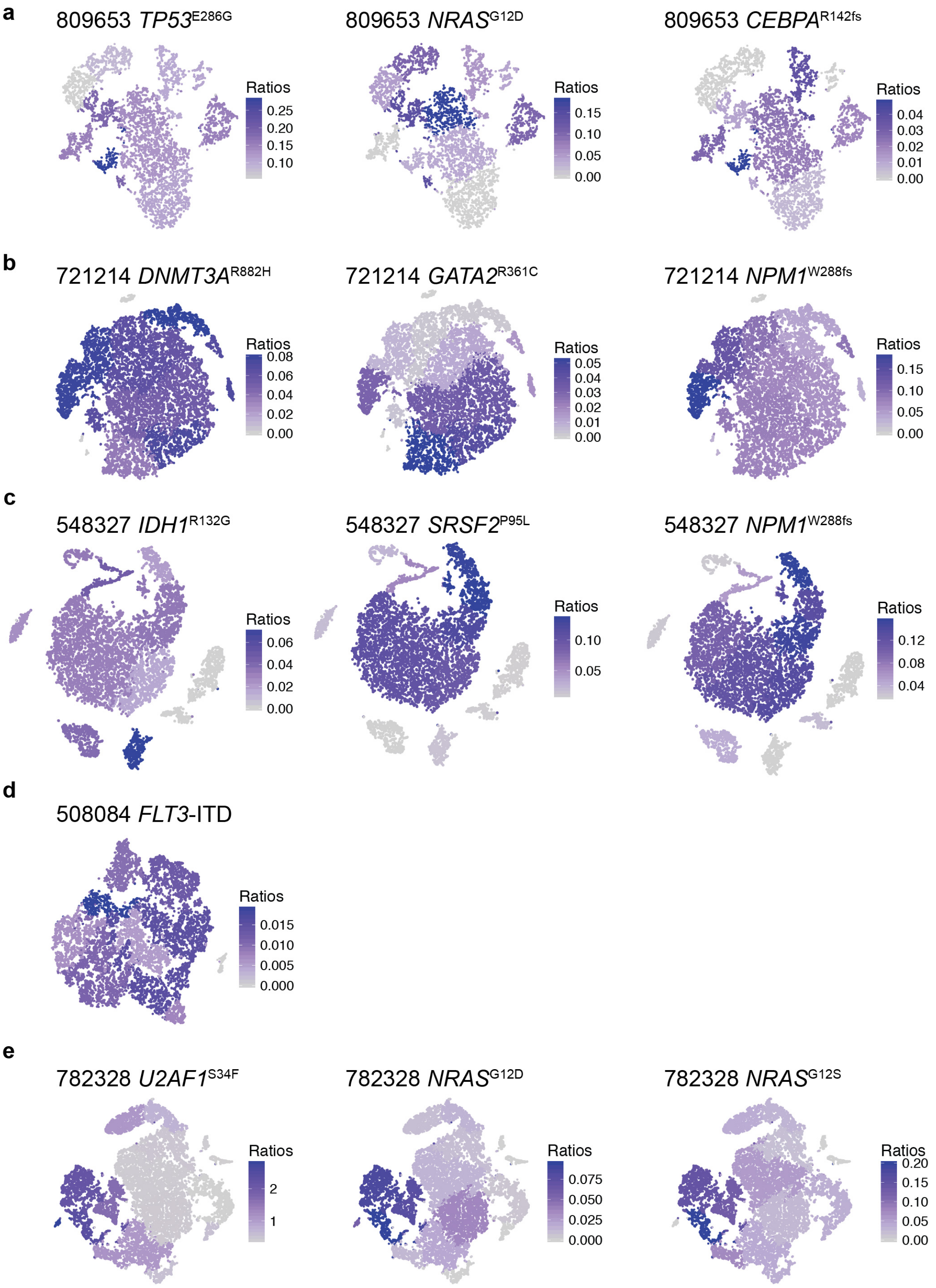
a
809653 TP53E286G
Ratios
0.25
0.20
0.15
0.10
809653 NRASG12D
Ratios
0.15
0.10
0.05
0.00
809653 CEBPAR142fs
Ratios
0.04
0.03
0.02
0.01
0.00
b
721214 DNMT3AR882H
Ratios
0.08
0.06
0.04
0.02
0.00
721214 GATA2R361C
Ratios
0.05
0.04
0.03
0.02
0.01
0.00
721214 NPM1W288fs
Ratios
0.15
0.10
0.05
0.00
c
548327 IDH1R132G
Ratios
0.06
0.04
0.02
0.00
548327 SRSF2P95L
Ratios
0.10
0.05
548327 NPM1W288fs
Ratios
0.12
0.08
0.04
d
508084 FLT3-ITD
Ratios
0.015
0.010
0.005
0.000
e
782328 U2AF1S34F
Ratios
2
1
782328 NRASG12D
Ratios
0.075
0.050
0.025
0.000
782328 NRASG12S
Ratios
0.20
0.15
0.10
0.05
0.00

Supplementary Figure 8. Clustered t-SNE plots colored according to expression-normalized mutation fraction in each cluster (selected genes). "Ratio" is the ratio of mutation fraction to average expression of the mutated gene. (a) 809653, putative AML cells only. (b) 721214. (c) 548327. (d) 508084. (e) 782328.

Supplementary Table 1. Representative functional enrichment of *GATA2*^{R361C}-associated genes

GO ID	GO Term	q-value	Selected genes
GO:0006955	immune response	4.49E-10	*CEBPB, HLA-DMB, HLA-DPA1, HLA-DPB1, HLA-DQA1, HLA-DQA2, CIITA*
GO:0012501	programmed cell death	1.68E-06	*HIF1A, HK1, CEBPB, KRAS, CAMK1D*
GO:0007159	leukocyte cell-cell adhesion	3.25E-06	*CEBPB, HLA-DMB, HLA-DPA1, HLA-DPB1, HLA-DQA1, HLA-DQA2*
GO:0005925	focal adhesion	2.30E-11	*AHNAK, FHL1, VIM, IRF2, GIT2, IQGAP1, PTPRC, KRAS*

Supplementary Table 2. Representative enrichment of *VIM*-correlated, subclone-specific genes.

GO ID	GO Term	q-value	Selected genes
GO:0006955	immune response	9.73E-06	*WAS, CDC42, CEBPB*
GO:0038094	Fc-gamma receptor signaling pathway	1.01E-04	*SYK, FGR, RAC1, WAS, ARPC5, ARPC1B, ARPC2, CDC42, HCK*
GO:0007010	cytoskeleton organization	8.42E-05	*VIM*
GO:0005925	focal adhesion	3.98E-09	*ITGB1BP1, IQGAP1, PTPRC, AHNAK, VIM*
N/A	WAS interactions	6.14E-05	*FGR, RAC1, WAS, GAS7, ARPC1B, ARPC2, RGS14, CDC42, HCK, PACSIN2*

Supplementary Table 3. Enrichment of genes that are more highly expressed in *RNF10* mutant-rich clusters.

GO ID	GO Term	q-value	Selected genes
GO:0032395	MHC class II receptor activity	6.25E-05	*HLA-DPA1, HLA-DQA1, HLA-DQB1, HLA-DQB2*
GO:0098609	cell-cell adhesion	9.63E-07	*SOX4, FLT3, ICAM3, ITGA4, CD34, CD47, CD74*
GO:0070489	T cell aggregation	1.08E-04	*SOX4, FLT3, HLA-DPA1, HLA-DPB1, HLA-DQA1, HLA-DQB1, HLA-DQB2, CD74, IL2RG, CDK6*
N/A	VCAM1 interactions	1.38E-10	*TUBB, STMN1, ITGA4, MSI2, HIST1H4C, RPLP0 (etc), RPS3 (etc), EEF1A1 (etc), EIF3L*
N/A	MCM2 interactions	1.05E-05	*SOX4, IMPDH2, TUBB, ITGA4, ADA, HIST1H4C, MACF1, CDK6, RPLP0 (etc), RPSA (etc), EIF3L, EEF1A1 (etc)*

N/A	NPM1 interactions	1.40E-05	*IMPDH2, CPSF6, RPLP0 (etc), RPS3 (etc), EEF1A1 (etc), EIF3L*

Supplementary Table 4. Enrichment of genes that are more highly expressed in *CEBPA* mutant-rich clusters.

GO ID	GO Term	q-value	Selected genes
GO:0003735	structural constituent of ribosome	9.95E-43	*RPL22, RPL23A, RPL26, etc.*
GO:0042110	T cell activation	1.25E-03	*HLA-DPA1, HLA-DPB1, HLA-DQB1, HLA-DRA, XBP1, CD74, TNFRSF4, FLT3, FOXP1, MYB, SOX4, KIT, RAC2, ZEB1, GSN, CDK6, RPL22, RPS6*
GO:0008134	transcription factor binding	5.84E-03	*MYC, GATA2, NPM1, TCF4, ZEB1, RUNX1, HLA-DQB1, XBP1, MEF2C, HOXA7, CD34, HDAC1*
N/A	MYC binding sites	9.22E-03	*FLT3, MYB, DNMT3A, NPM1, TCF4, SET, STMN1, HHEX, MAX, HOXA7, HOXA9, RPL22 (etc), RPS19 (etc), EIF3E (etc)*
