# Supplementary material for: A general approach for detecting expressed mutations in AML cells using single cell RNA-sequencing
Source: Nat Commun. 2019 Aug 14;10:3660. doi: 10.1038/s41467-019-11591-1 (PMC6694122; doi:10.1038/s41467-019-11591-1)
Supplement: Supplementary file 2 — Description of Additional Supplementary Files [file 41467_2019_11591_MOESM2_ESM.pdf]

Description of Additional Supplementary Files

File Name: Supplementary Data 1
Description: Table of somatic variants discovered using eWGS, including variant allele frequencies (VAFs) in eWGS, bulk RNA-seq, and scRNA-seq data, as well as Mutant Cell Detection Rate (and related statistics), subclone assignments, and lists of mutation-containing cells.

File Name: Supplementary Data 2
Description: Summary coverage metrics for 200 cancer-relevant genes.

File Name: Supplementary Data 3
Description: Somatic variant false positive rates computed from control AML samples.

File Name: Supplementary Data 4
Description: Normalized expression data used to generate heatmaps for each case (Fig. 2, Supp. Fig. 2-5).

File Name: Supplementary Data 5
Description: Differentially expressed genes for (a) genes whose expression is correlated with the *GATA2*^{R361C} subclone, calculated using all putative AML cells, (b) genes whose expression is correlated with *GATA2*^{R361C} itself, calculated using all putative AML cells (c) genes whose expression is correlated with the *GATA2*^{R361C} subclone, calculated using mutant cells, and (d) 198 genes in the putative *VIM* regulon represented in Fig. 5h.
