# Supplementary material for: A general approach for detecting expressed mutations in AML cells using single cell RNA-sequencing
Source: Nat Commun. 2019 Aug 14;10:3660. doi: 10.1038/s41467-019-11591-1 (PMC6694122; doi:10.1038/s41467-019-11591-1)
Supplement: Supplementary file 8 — Reporting Summary [file 41467_2019_11591_MOESM8_ESM.pdf]

nature research

Corresponding author(s): Timothy (J.) Ley

Reporting Summary

Nature Research wishes to improve the reproducibility of the work that we publish. This form provides structure for consistency and transparency in reporting. For further information on Nature Research policies, see Authors & Referees and the Editorial Policy Checklist.

Statistical parameters

When statistical analyses are reported, confirm that the following items are present in the relevant location (e.g. figure legend, table legend, main text, or Methods section).

n/a	Confirmed	
☒	☐	The exact sample size (n) for each experimental group/condition, given as a discrete number and unit of measurement
☒	☐	An indication of whether measurements were taken from distinct samples or whether the same sample was measured repeatedly
☐	☒	The statistical test(s) used AND whether they are one- or two-sided<br>*Only common tests should be described solely by name; describe more complex techniques in the Methods section.*
☐	☒	A description of all covariates tested
☐	☒	A description of any assumptions or corrections, such as tests of normality and adjustment for multiple comparisons
☐	☒	A full description of the statistics including central tendency (e.g. means) or other basic estimates (e.g. regression coefficient) AND variation (e.g. standard deviation) or associated estimates of uncertainty (e.g. confidence intervals)
☒	☐	For null hypothesis testing, the test statistic (e.g. F, t, r) with confidence intervals, effect sizes, degrees of freedom and P value noted<br>*Give P values as exact values whenever suitable.*
☒	☐	For Bayesian analysis, information on the choice of priors and Markov chain Monte Carlo settings
☒	☐	For hierarchical and complex designs, identification of the appropriate level for tests and full reporting of outcomes
☐	☒	Estimates of effect sizes (e.g. Cohen's d, Pearson's r), indicating how they were calculated
☒	☐	Clearly defined error bars<br>*State explicitly what error bars represent (e.g. SD, SE, CI)*

Our web collection on statistics for biologists may be useful.

Software and code

Policy information about availability of computer code

Data collection	No software was used in data collection.
Data analysis	Data analysis was performed using the following software: BWA-MEM 0.7.10, Picard 1.113, Samtools r982, Somatic Sniper 1.0.4, VarScan 2.3.6, Strelka 1.0.11, Mutect 1.1.4, GATK somatic-indel 5336, Pindel 0.5, CopyCat 1.6.10, Lumpy 0.2.610, GATK HaplotypeCaller 3.5, Manta 0.29, FastQC, SciClone 1.1, Kallisto 0.43.1, the UCSC Genome Browser, bamCoverage from deepTools 3.1.3, Gviz 1.22.3, BiomaRt 2.34.2, CONICSmat, ToppFun (https://toppgene.cchmc.org/enrichment.jsp), Cell Ranger 2.1.1, Seurat 2.2.1, R 3.4.0, and custom Pysam-based tools available at https://github.com/sridnona/cb_sniffer and https://github.com/genome/scrna_mutations/tree/master/misc_scripts.

For manuscripts utilizing custom algorithms or software that are central to the research but not yet described in published literature, software must be made available to editors/reviewers upon request. We strongly encourage code deposition in a community repository (e.g. GitHub). See the Nature Research guidelines for submitting code & software for further information.

Data

Policy information about availability of data

All manuscripts must include a data availability statement. This statement should provide the following information, where applicable:

- Accession codes, unique identifiers, or web links for publicly available datasets
- A list of figures that have associated raw data
- A description of any restrictions on data availability

Enhanced whole genome sequence (eWGS), bulk RNA-sequence, and single cell RNA-sequence (scRNA-seq) data generated during the current study are available in dbGaP (https://www.ncbi.nlm.nih.gov/gap/) with the primary accession code phs000159. The SRA IDs for this study are: SRR7904017, SRR7904018, SRR7904019, SRR7904020, SRR7910353, SRR7910351, SRR7910349, SRR7904016, SRR7903979, SRR7825447, SRR7825459, SRR7825446, SRR7825444, SRR7825491, SRR7825473, SRR7825453, SRR7825466, SRR7825499, SRR7825482, and SRR7939318.

Field-specific reporting

Please select the best fit for your research. If you are not sure, read the appropriate sections before making your selection.

☒ Life sciences ☐ Behavioural & social sciences ☐ Ecological, evolutionary & environmental sciences

For a reference copy of the document with all sections, see nature.com/authors/policies/ReportingSummary-flat.pdf

Life sciences study design

All studies must disclose on these points even when the disclosure is negative.

Sample size	Five samples, representing five unique tumors, were analyzed in order to evaluate our approach in genetically representative tumors. For each sample, we sequenced up to 20,000 cells, a number determined by technological feasibility. The conclusions of this study were based on the consistent performance of our approach in each of the five samples.
Data exclusions	No data were excluded.
Replication	We demonstrated the feasibility of our approach in each of five samples. Each sample represents a unique tumor, and the exact results are tumor-specific.
Randomization	Randomization was not relevant to this study. All samples were analyzed the same way, and there were no treatment groups or inter-sample comparisons.
Blinding	Blinding was not relevant to this study. No treatments were applied to the samples, and no inter-sample comparisons were performed.

Reporting for specific materials, systems and methods

Materials & experimental systems

n/a	Involved in the study	
☐	☒	Unique biological materials
☐	☒	Antibodies
☒	☐	Eukaryotic cell lines
☒	☐	Palaeontology
☒	☐	Animals and other organisms
☒	☐	Human research participants

Methods

n/a	Involved in the study	
☒	☐	ChIP-seq
☐	☒	Flow cytometry
☒	☐	MRI-based neuroimaging

Unique biological materials

Policy information about availability of materials

Obtaining unique materials	This study used cryopreserved AML samples from now-deceased individuals. Additional cryovials may not be available in every case.

Antibodies

Antibodies used	CD99-FITC (ThermoFisher, clone 3B2/TA8)
Validation	Mouse isotype antibody was used as a negative staining reference (eBioscience, clone eBMG2b). The antibody was "pre-titrated and tested by flow cytometric analysis of normal human peripheral blood cells" by ThermoFisher.

Flow Cytometry

Plots

Confirm that:

☒ The axis labels state the marker and fluorochrome used (e.g. CD4-FITC).

☒ The axis scales are clearly visible. Include numbers along axes only for bottom left plot of group (a 'group' is an analysis of identical markers).

☒ All plots are contour plots with outliers or pseudocolor plots.

☒ A numerical value for number of cells or percentage (with statistics) is provided.

Methodology

Sample preparation	Cryopreserved primary human AML tumors were thawed, washed with phosphate buffered saline, and stained with CD99-FITC (ThermoFisher, clone 3B2/TA8) for 30 minutes at 4C in PBS with 2% FBS and 0.25mM EDTA. Samples were stained with SYTOX-Blue (Thermo-Fisher) for viability.
Instrument	Sorting was performed on a modified Sony Synergy SY3200 (Sony Biotechnology, San Jose, CA) updated to 24 parameters.
Software	Software used for acquisition was Winlist version 8 (Verity Software House, Topsham, ME)
Cell population abundance	5 million viable CD99-FITC stained human AML cells with a recovery of 724k CD99-low and 800k CD99-high cells
Gating strategy	Single, viable cells were gated to collect the top and bottom 15% of events by CD99-FITC staining intensity.

☒ Tick this box to confirm that a figure exemplifying the gating strategy is provided in the Supplementary Information.
